# Supplementary material for: Calcium-dependent ESCRT recruitment and lysosome exocytosis maintain epithelial integrity during Candida albicans invasion
Source: Cell Rep. 2022 Jan 4;38(1):110187. doi: 10.1016/j.celrep.2021.110187 (PMC8755444; doi:10.1016/j.celrep.2021.110187)
Supplement: Document S1. Figures S1–S4 [file mmc1.pdf]

Cell Reports, Volume *38*

Supplemental information

Calcium-dependent ESCRT recruitment and lysosome exocytosis maintain epithelial integrity during *Candida albicans* invasion

Johannes Westman, Jonathan Plumb, Anna Licht, Mabel Yang, Stefanie Allert, Julian R. Naglik, Bernhard Hube, Sergio Grinstein, and Michelle E. Maxson

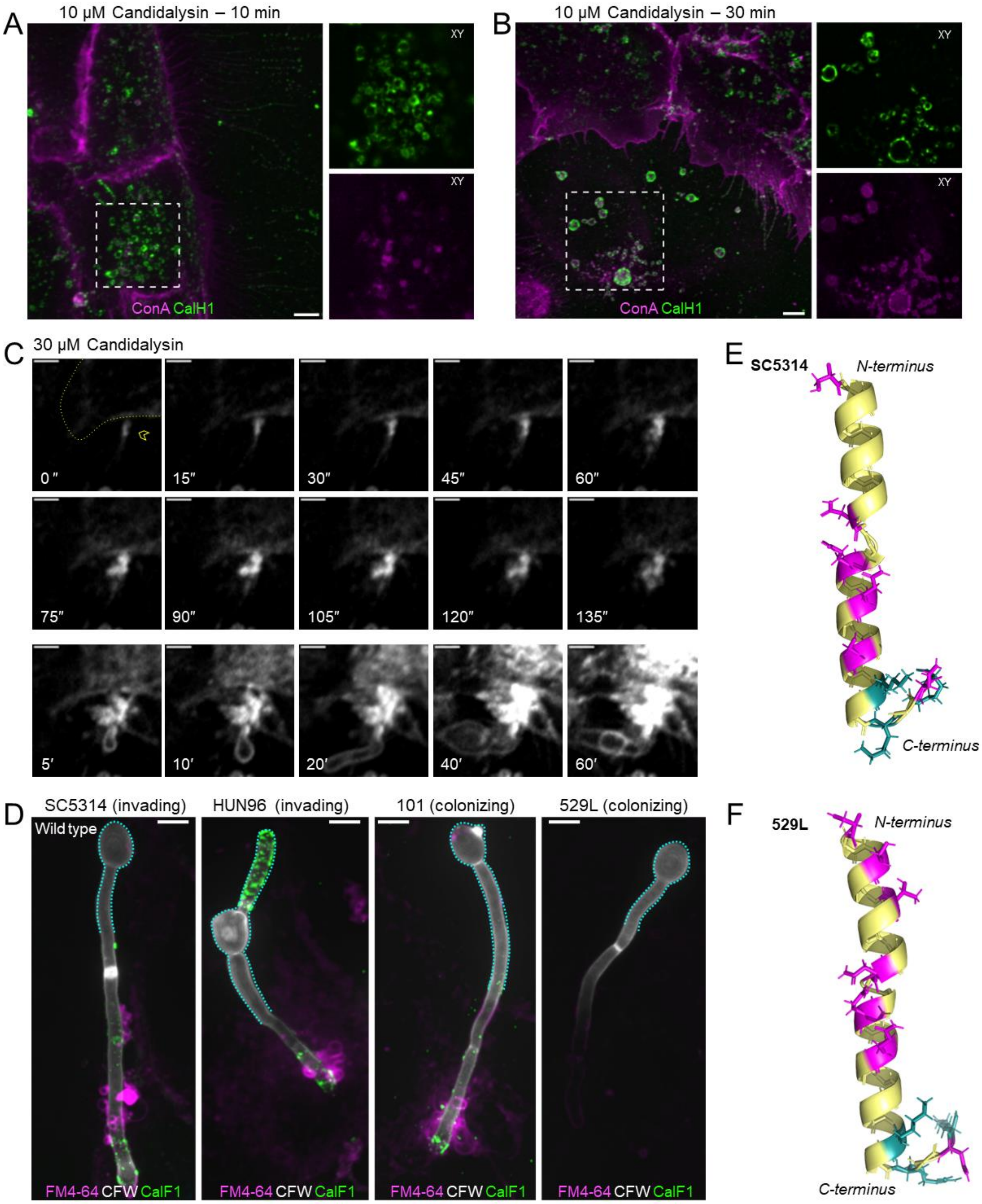
A
10 µM Candidalysin – 10 min
XY
XY
ConA CalH1
B
10 µM Candidalysin – 30 min
XY
XY
ConA CalH1
C
30 µM Candidalysin
0″
15″
30″
45″
60″
75″
90″
105″
120″
135″
5′
10′
20′
40′
60′
D
SC5314 (invading)
HUN96 (invading)
101 (colonizing)
529L (colonizing)
Wild type
FM4-64 CFW CalF1
FM4-64 CFW CalF1
FM4-64 CFW CalF1
FM4-64 CFW CalF1
E
SC5314
N-terminus
C-terminus
F
529L
N-terminus
C-terminus

Figure S1. Candidalysin induces epithelial plasma membrane blebbing, related to Figure 4.

Confocal micrographs of TR146 treated with 10 µM candidalysin peptide for (A) 10 min or (B) 30 min. After treatments, non-permeablized cells were stained for the PM and PM-derived blebs/debris using ConA, and for the localization of candidalysin using the CaLH1 nanobody. Side panels are *XY* slices showing individual CalF1 (green) and ConA (magenta) channels, for areas marked by the dotted boxes. (C) TR146 treated with incubated with 20 µM FM4-64 and cell Z-stacks imaged by time-lapse microscopy. After 1 min of imaging, 30 µM candidalysin peptide was added, and cells imaged for a total time of 90 min. Cell plasma membrane and location of bleb formation is marked in the first panel by the dotted yellow line and yellow arrowhead, respectively. Panels are extended focus projections. (D) Representative confocal micrographs of TR146 invasion pockets induced by *C. albicans* clinical isolates SC5314, HUN96, 101 and 529L. Bleb formation and candidalysin was assessed by staining non-permeabilized cells with 10 µM FM4-64 and CaLF1 nanobody, respectively. External portions of the isolates, stained with ConA, are indicated by the dotted cyan outlines. All scale bars = 5 µm. Images are representative of at least 3 experiments of each type. Structure predictions for the candidalysin peptides of isolates (E) SC5314 and (F) 529L. Amino acid residues were colored in PyMOL as follows: Hydrophobic– yellow, polar– magenta, and charged– teal.

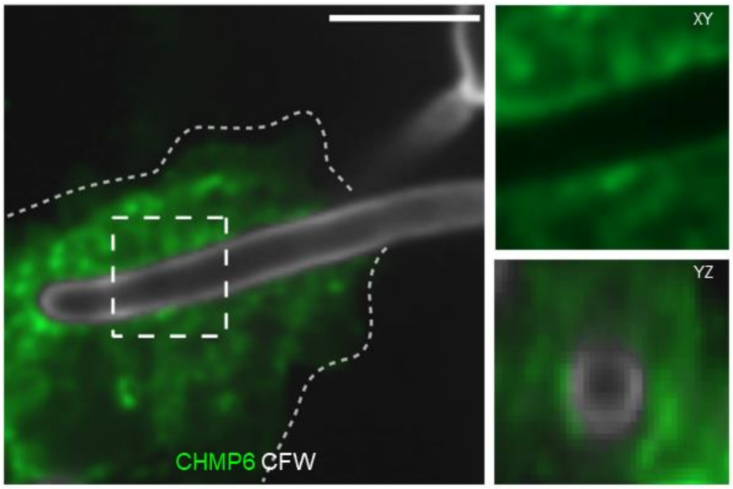

Figure S2. CHMP6 localizes to the invasion pocket membrane, related to Figure 5.

(A) The invasion pocket recruits ESCRT-III component CHMP6. Confocal micrograph of invasion pockets containing wild-type *C. albicans.* TR146 cells were transfected with CHMP6-GFP. Side panels show the XY and YZ slices corresponding to the areas marked by the dashed boxes. Outlines of TR146 cells are indicated by dotted lines. Scale bars = 5 µm.

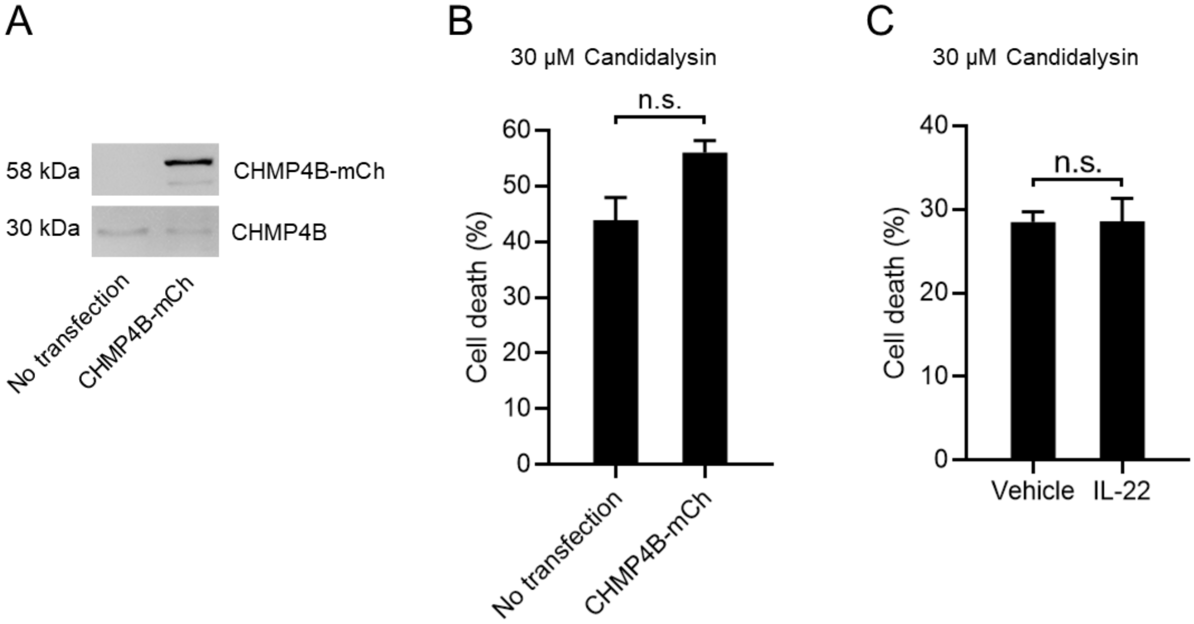

Figure S3. Overexpression of the ESCRT protein CHMP4B does not improve cell viability after treatment with candidalysin, related to Figure 6.

(A) CHMP4B-mCherry was transiently overexpressed in TR146 cell using Lipofectamine LTX. The protein expression of endogenous CHMP4B and CHMP4B-mCherry was measured using different exposure times on the same Western blotting membrane. (B) Quantification of cell death in CHMP4B-mCh transfected TR146 cells and in non-transfected cell. Data are means ± SEM of 3 individual experiments. (C) Human recombinant IL-22 does not improve cell viability after treatment with candidalysin.TR146 cells were incubated with human recombinant 100ng/mL IL-22 for 48 h prior to a treatment with 30 μM candidalysin for 1 h. Cells were incubated with propidium iodide to identify dead cells, fixed in 4% PFA and permeabilized with 0.2% TritonX-100. Lastly, cells were counter-stained with SYTOX Green to visualize all nuclei. Data are means ± SEM of 3 individual experiments.

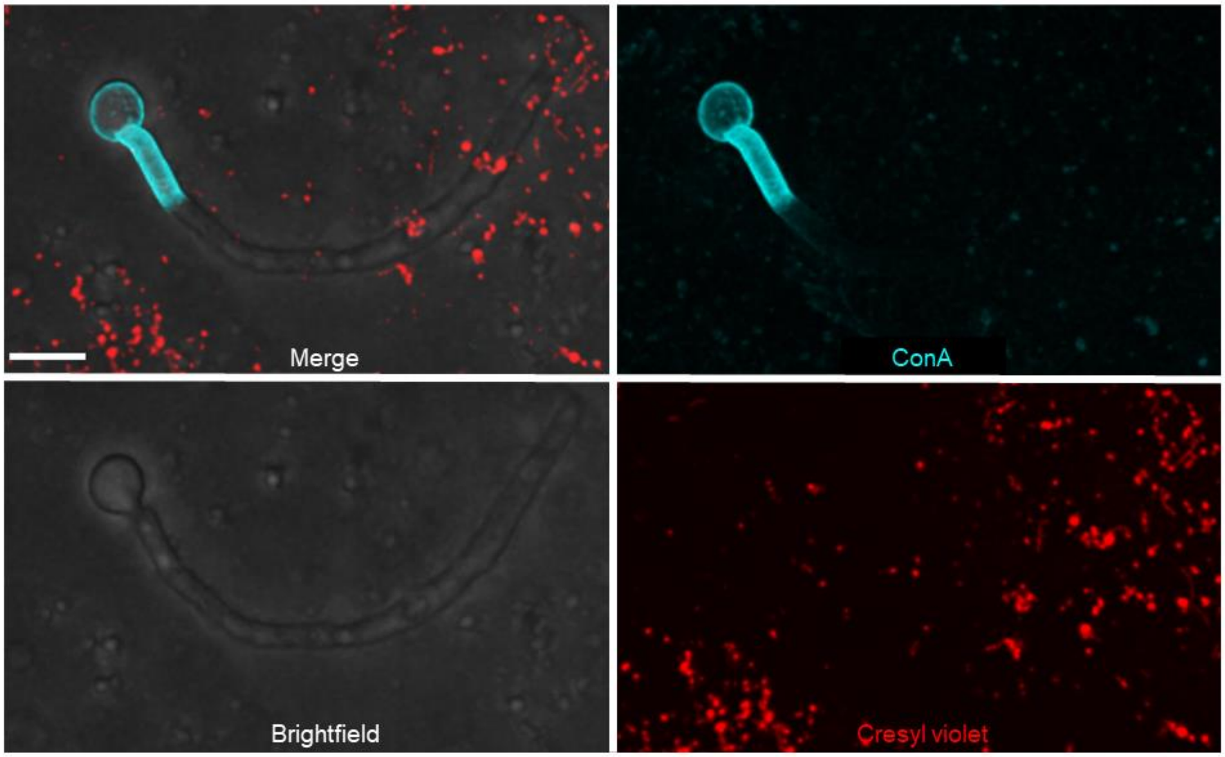

Figure S4. Invasion pockets do not retain the acidotropic dye cresyl violet, related to Figure 7.

The acidity of *C. albicans*-containing invasion pockets was assessed by incubating TR146 cells infected with the wild-type fungus with cresyl violet (red) for 2 min prior to spinning-disk confocal imaging. Extracellular portions of the invading *C. albicans* were stained with ConA (cyan). Scale bar = 5 µm.
