# Supplementary material for: Calcium-dependent ESCRT recruitment and lysosome exocytosis maintain epithelial integrity during Candida albicans invasion
Source: Cell Rep. 2022 Jan 4;38(1):110187. doi: 10.1016/j.celrep.2021.110187 (PMC8755444; doi:10.1016/j.celrep.2021.110187)
Supplement: Document S2. Article plus supplemental information [file mmc5.pdf]

Cell Reports

Article

Calcium-dependent ESCRT recruitment and lysosome exocytosis maintain epithelial integrity during *Candida albicans* invasion

Graphical abstract

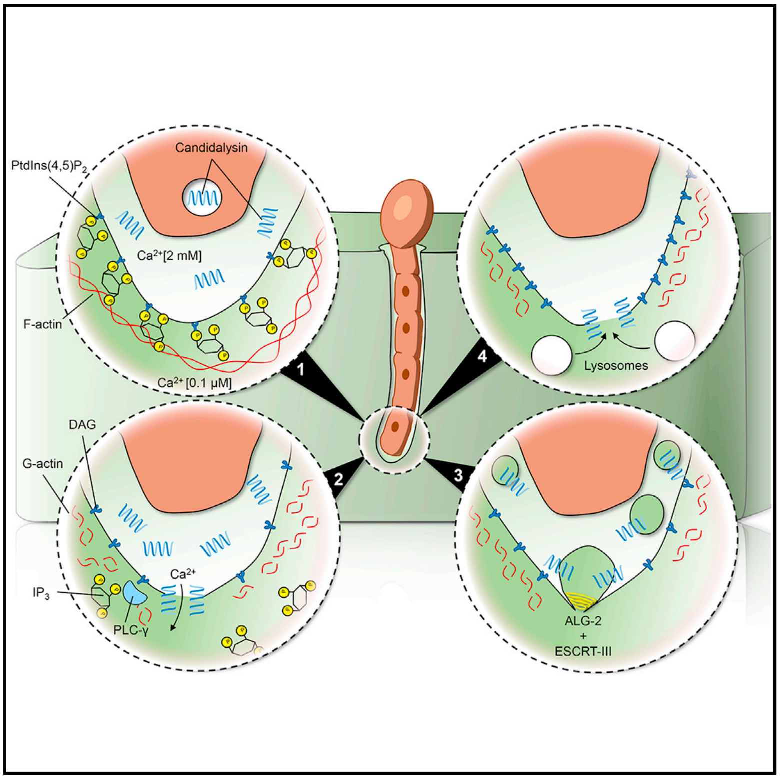

Highlights

- *C. albicans* invasion and candidalysin secretion trigger epithelial responses
- Candidalysin causes Ca^{2+} influx, PtdIns(4,5)P_2 hydrolysis, and breakdown of F-actin
- ESCRT and exocytosis of lysosomes repair the host membrane and remove candidalysin
- Silencing the ESCRT machinery curtails the viability of candidalysin-treated cells

Authors

Johannes Westman, Jonathan Plumb, Anna Licht, ..., Bernhard Hube, Sergio Grinstein, Michelle E. Maxson

Correspondence

bernhard.hube@hki-jena.de (B.H.), sergio.grinstein@sickkids.ca (S.G.)

In brief

The human fungal pathogen *Candida albicans* invades epithelial cells and secretes the pore-forming toxin candidalysin. Westman et al. demonstrate that epithelial cells implement two membrane repair mechanisms—ESCRT-mediated plasma membrane pinching and lysosome exocytosis—that remove candidalysin and confer resistance to the invading fungus

Westman et al., 2022, Cell Reports *38*, 110187
January 4, 2022 © 2021 The Authors.
https://doi.org/10.1016/j.celrep.2021.110187

CellPress

Cell Reports

CellPress
OPEN ACCESS

Article

Calcium-dependent ESCRT recruitment and lysosome exocytosis maintain epithelial integrity during *Candida albicans* invasion

Johannes Westman,[1] Jonathan Plumb,[1] Anna Licht,[1] Mabel Yang,[1] Stefanie Allert,[2] Julian R. Naglik,[3] Bernhard Hube,[2,4,*] Sergio Grinstein,[1,5,6,7,*] and Michelle E. Maxson[1]

[1]Program in Cell Biology, The Hospital for Sick Children, Toronto, ON M5G 0A4, Canada

[2]Department of Microbial Pathogenicity Mechanisms, Leibniz Institute for Natural Product Research and Infection Biology-Hans Knöll Institute (HKI), 07745 Jena, Germany

[3]Centre for Host-Microbiome Interactions, Faculty of Dentistry, Oral and Craniofacial Sciences, King's College London, London SE1 9RT, UK

[4]Institute of Microbiology, Friedrich Schiller University, 07745 Jena, Germany

[5]Department of Biochemistry, University of Toronto, Toronto, ON M5S 1A8, Canada

[6]Keenan Research Centre for Biomedical Science, St. Michael's Hospital, Toronto, ON M5C 1N8, Canada

[7]Lead contact

*Correspondence: bernhard.hube@hki-jena.de (B.H.), sergio.grinstein@sickkids.ca (S.G.)

https://doi.org/10.1016/j.celrep.2021.110187

SUMMARY

Candida albicans is both a commensal and an opportunistic fungal pathogen. Invading hyphae of *C. albicans* secrete candidalysin, a pore-forming peptide toxin. To prevent cell death, epithelial cells must protect themselves from direct damage induced by candidalysin and by the mechanical forces exerted by expanding hyphae. We identify two key Ca^{2+}-dependent repair mechanisms employed by epithelial cells to withstand candidalysin-producing hyphae. Using camelid nanobodies, we demonstrate candidalysin secretion directly into the invasion pockets induced by elongating *C. albicans* hyphae. The toxin induces oscillatory increases in cytosolic $[Ca^{2+}]$, which cause hydrolysis of PtdIns(4,5)P_2 and loss of cortical actin. Epithelial cells dispose of damaged membrane regions containing candidalysin by an Alg-2/Alix/ESCRT-III-dependent blebbing process. At later stages, plasmalemmal tears induced mechanically by invading hyphae are repaired by exocytic insertion of lysosomal membranes. These two repair mechanisms maintain epithelial integrity and prevent mucosal damage during both commensal growth and infection by *C. albicans.*

INTRODUCTION

Candida albicans colonizes the mucosal surfaces of the gut, oral cavity, or vaginal tracts. The fungus is a commensal constituent of the microbiome of most healthy individuals. A balanced microbiome and an intact immune system are required to maintain the fungus in the commensal state, preventing invasion, epithelial damage, and mucosal infection. Accordingly, antibiotic treatments and immunosuppression are predisposing factors for *C. albicans* infection (Kumamoto et al., 2020).

Superficial *C. albicans* mucosal infections are common. However, in severe cases, such as in immunocompromized patients, invasion and translocation from the gut enable the fungus to disseminate via the blood stream to internal organs, causing life-threatening infections (Perlroth et al., 2007; Pfaller and Diekema, 2007; Mayer et al., 2013). A key aspect of this commensal-to-pathogen shift is the fungal morphology. *C. albicans* exists primarily in yeast form, but it can also adopt filamentous hyphal forms. Hyphae, which can grow several microns per hour, can invade epithelia forming an invasion pocket (Moyes et al., 2015). Importantly, hyphal formation per se is not sufficient to cause epithelial damage (Moyes et al., 2016). Rather, a combination of hyphal formation and extension, and the expression of hyphal-associated genes is essential to damage epithelial cells (Westman et al., 2019; Mogavero et al., 2021). In particular, the hypha-associated peptide toxin candidalysin encoded by the *ECE1* gene is essential to cause damage (Moyes et al., 2016; Allert et al., 2018; Naglik et al., 2019). However, the presence of *C. albicans* hyphae is not necessarily associated with infection, and moderate levels of invading hyphae are tolerated (Moyes et al., 2010). How epithelial cells preserve their integrity when challenged by growing hyphae and by release of the pore-forming candidalysin remains unknown. The extending hyphae can burrow into the mucosal cells, inflicting severe mechanical stress, while concomitantly membrane permeabilization by candidalysin disrupts the cellular ionic and osmotic balance. It is remarkable that mucosal integrity is maintained when continuously confronted by these challenges.

We sought to understand the manner whereby epithelial cells overcome the stresses imposed by growing *C. albicans* hyphae. We report that when confronted by growing hyphae, epithelial cells enable a combination of protective mechanisms that repair mechanical damage to the membrane, eliminate candidalysin, and restore ionic homeostasis.

 © 2021 The Authors.
This is an open access article under the CC BY license (http://creativecommons.org/licenses/by/4.0/).

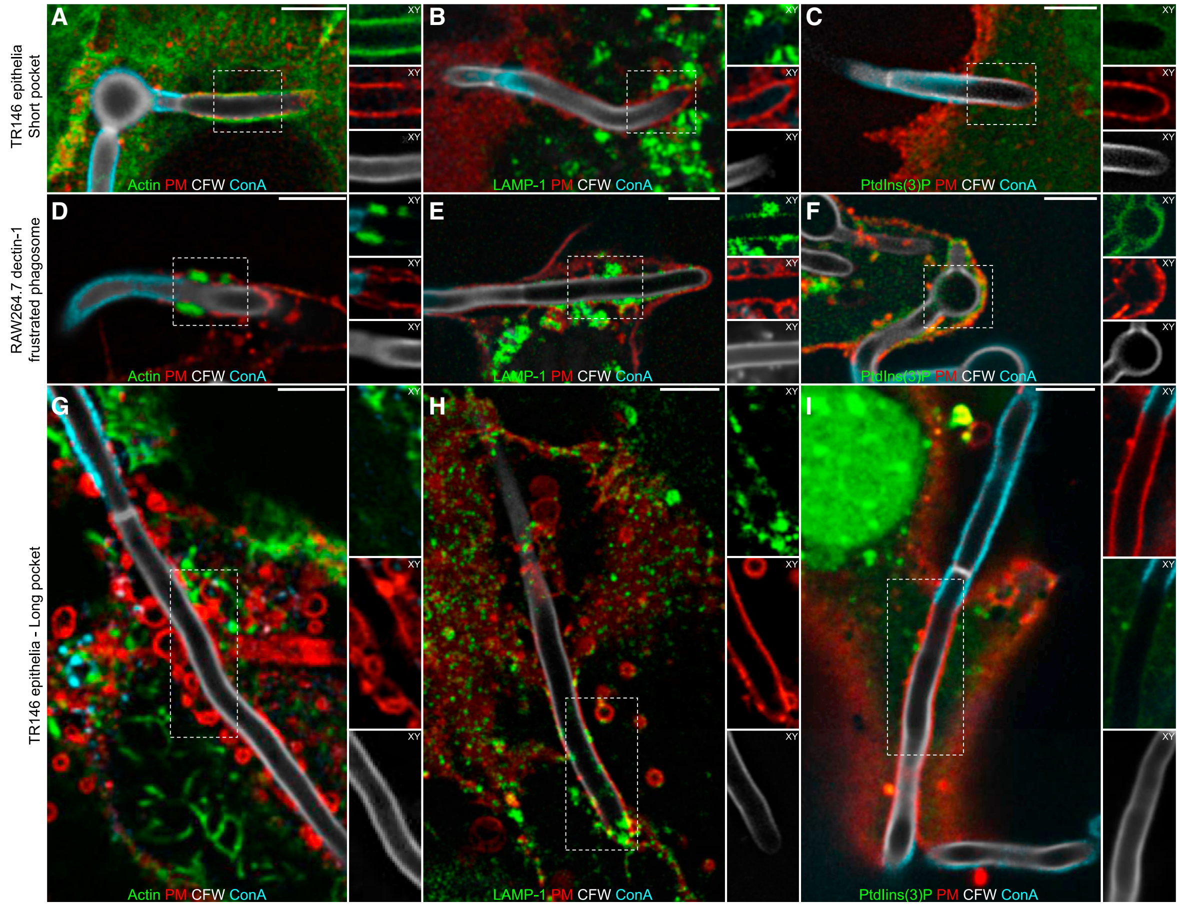

Figure 1. Characterization of the *C. albicans*-containing invasion pocket

(A–C) Confocal micrographs of short TR146 invasion pockets induced by wild-type *C. albicans*. In these and subsequent images the entire *C. albicans* was stained with calcofluor white (CFW) and those portions of the fungus outside the invasion pocket with concanavalin A (ConA). The PM was identified by expression of PM-RFP. Here and in all subsequent images, *actin* refers specifically to F-actin, which was stained with phalloidin (A); LAMP-1 immunostaining (B); PtdIns(3)P detected by expression of PX-GFP (C).

(D–F) Micrographs of RAW264.7 dectin-1 macrophages with frustrated phagosomes containing *C. albicans*. Micrographs show F-actin (D); LAMP-1 (E); PtdIns(3) P (F).

(G–I) Micrographs of long TR146 invasion pockets containing wild-type *C. albicans*. Micrographs show F-actin (G); LAMP-1 (H); PtdIns(3)P (I). Images are representative of $\geq$3 experiments of each type. All scale bars represent 5 μm.

RESULTS

Characterization of the *C. albicans*-containing invasion pocket

When growing on epithelial monolayers, *C. albicans* hyphae depress the host cell surface, generating an invasion pocket reminiscent of the deformations observed in infected primary tissues (Zakikhany et al., 2007; Dalle et al., 2010). To study the consequences of such deformation, we grew *C. albicans* for up to 6 h on TR146 cells, an immortalized line of oral epithelial origin. Invasion pockets generated by extending hyphae could be identified by visualizing the entire fungus with calcofluor white (CFW), while staining exposed regions of the cell wall with Alexa Fluor-conjugated concanavalin A (ConA); the lectin is excluded from invasion pockets by the tight adherence of the fungal wall to the invaginated host membrane (Figure 1). Short invasion pockets (<20 μm in length) had no discernible effect on the host cell plasma membrane (PM), which retained its continuity (assessed with the fluorescent plasmalemmal probe PM-RFP) and maintained an intact layer of cortical F-actin (stained with phalloidin) (Figure 1A). Of note, short pockets were devoid of PtdIns(3)P (detected using PX-GFP) and of LAMP-1 (visualized expressing LAMP-1-GFP), implying that neither early endosomes nor late endosomes/lysosomes had fused with the invaginated PM (Figures 1B and 1C). This contrasts with the invaginations formed in macrophages around similarly sized hyphae. As reported (Maxson et al., 2018), the structures formed by macrophages are akin to frustrated phagosomes, characterized by a thick actin cuff at the neck (Figure 1D) that enables retention of PtdIns(3)P and late endosomal/lysosomal markers that fuse with the limiting membrane (Figures 1E and 1F), yet devoid of actin along the rest of the invaginated membrane.

As the fungus continued to grow into the epithelial cell, the appearance of the invasion pocket was markedly altered. Long

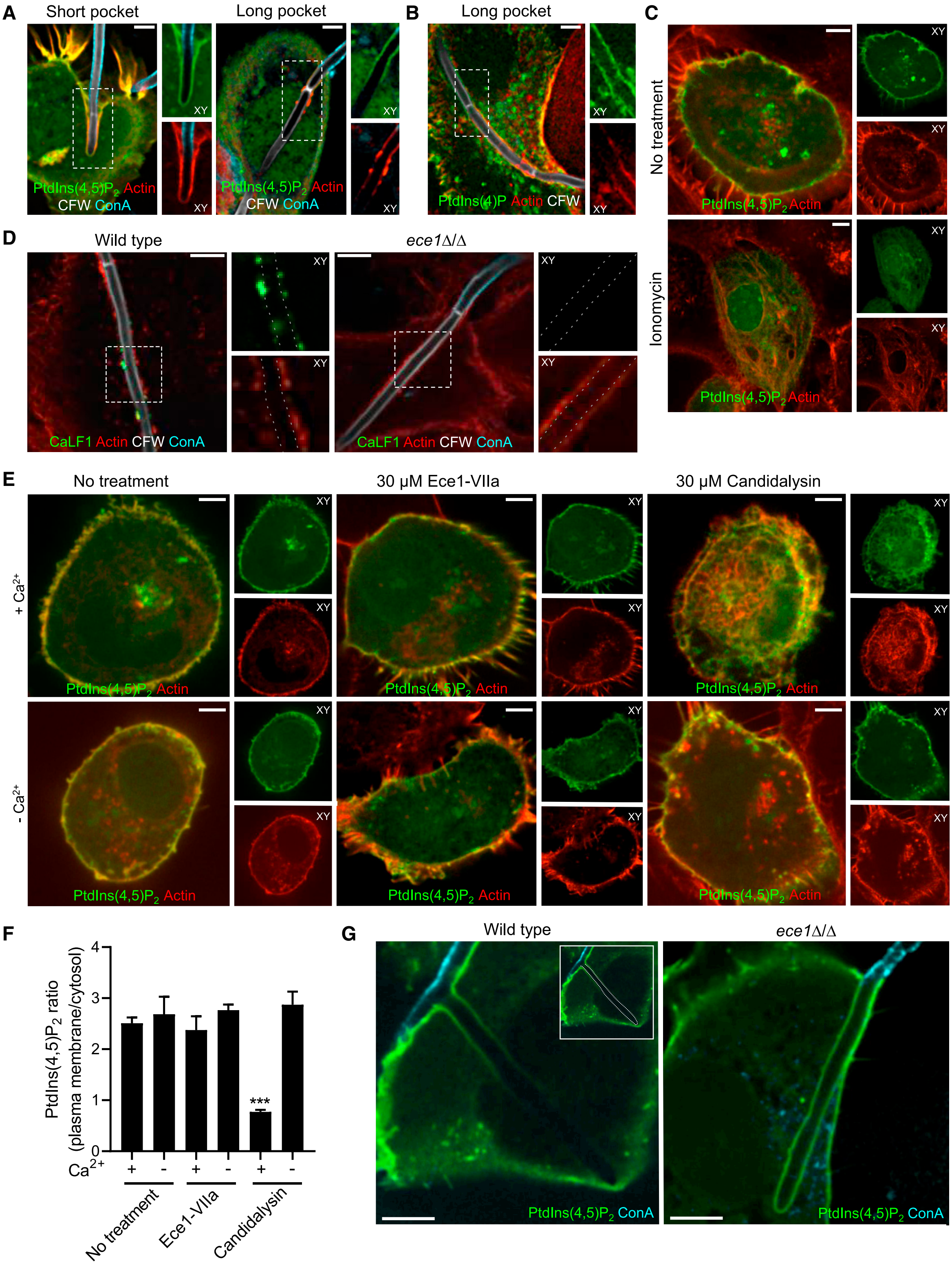

Figure 2. Damage to the invasion pocket leads to influx of extracellular Ca^{2+}, hydrolysis of PtdIns(4,5)P_2, and loss of cortical actin

(A) Confocal micrograph of short invasion pockets containing *C. albicans*. TR146 cells were transfected with the PtdIns(4,5)P_2-binding probe PLCδ-GFP and stained as in Figure 1.

(B) Micrograph of a long invasion pocket. TR146 cells transfected with the PtdIns(4)P-binding probe 2xP4M-GFP.

(legend continued on next page)

(>20 μm) pockets showed progressive loss of cortical actin, PM markers showed discontinuities (Figure 1G), and lysosome insertion became apparent (Figure 1H). We found no evidence of early endosome fusion, as pockets remained negative for PtdIns(3)P (Figure 1I). The patchy depolymerization of cortical actin (Figure 1G) was suggestive of significant damage to long invasion pockets.

Damage to the invasion pocket leads to influx of extracellular Ca^{2+}, hydrolysis of PtdIns(4,5)P_2, and loss of cortical actin

The mechanism that leads to the apparent PM damage was investigated next. Cortical actin is stabilized in part by ezrin, radixin, and moesin proteins through interactions with PtdIns(4,5)P_2. We therefore tested whether depletion of PtdIns(4,5)P_2 accompanies the loss of cortical actin. The distribution of the phosphoinositide was visualized by expressing the pleckstrin homology (PH) domain of phospholipase C-δ (PH-PLCδ). Short invasion pockets retained PtdIns(4,5)P_2 along with cortical actin (Figure 2A). In contrast, a loss of plasmalemmal PtdIns(4,5)P_2 was evident in long pockets that displayed discontinuous cortical actin (Figure 2A). As shown in Figure 2B, the depletion of PtdIns(4,5)P_2 was not attributable to loss of PtdIns(4)P, the substrate required for its synthesis. Instead, it appeared more likely that accelerated degradation was responsible for PtdIns(4,5)P_2 loss.

Activation of phospholipase C by Ca^{2+} is often responsible for the hydrolysis of PtdIns(4,5)P_2. As shown in Figure 2C, elevation of cytosolic Ca^{2+} ($[Ca^{2+}]_c$) by means of ionomycin also effectively depleted plasmalemmal PtdIns(4,5)P_2. Importantly, the depletion of PtdIns(4,5)P_2 induced by ionomycin was accompanied by loss of cortical F-actin, which depolymerized and redistributed to intracellular structures (Figure 2C). This raised the possibility that changes in $[Ca^{2+}]_c$ were responsible for the alterations caused by deeply penetrating hyphae. Such changes could in principle be inflicted mechanically or, more likely, by the release and accumulation of candidalysin—a pore-forming toxin—in the pocket. To evaluate the latter possibility, we used two α-candidalysin nanobodies conjugated to Alexa Fluor dyes (Mogavero et al., 2021). Of note, the nanobodies were sufficiently small to enter invasion pockets without the need to permeabilize cells after fixation, enabling us to distinguish secreted candidalysin from that still retained inside *C. albicans'* secretory organelles. In cells infected with wild-type *C. albicans* the nanobodies could consistently detect candidalysin inside invasion pockets (Figure 2D). Candidalysin often accumulated at sites of cortical actin breakdown, suggesting that the toxin is responsible for the breakdown (Figure 2D). In contrast, invasion pockets containing the candidalysin-deficient *ece1*Δ/Δ mutant failed to show immunostaining, confirming the specificity of the nanobodies (Figure 2D). Importantly, actin integrity was largely maintained along the invasion pockets formed by candidalysin-deficient hyphae, implying that the toxin is responsible for the effects caused by the wild-type *C. albicans* (Figure 2D).

To directly assess the ability of candidalysin to alter PtdIns(4,5)P_2 and cortical actin, TR146 cells were treated with pure toxin generated synthetically; for comparison, parallel samples were treated with a synthetic version of Ece1-VIIa, another peptide derived from the Ece1 polypeptide that lacks the pore-forming properties ascribed to candidalysin (Moyes et al., 2016). As shown in Figure 2E, addition of candidalysin sufficed to deplete PtdIns(4,5)P_2 and F-actin from the PM, while peptide Ece1-VIIa was without effect. The effect of candidalysin required extracellular Ca^{2+} (*cf.* top right and bottom right panels in Figure 2E). The effects of the peptides on PtdIns(4,5)P_2 are quantified in Figure 2F. Together, these findings suggest that candidalysin depletes plasmalemmal PtdIns(4,5)P_2—and consequently also cortical actin—by increasing the permeability of the PM to extracellular Ca^{2+}.

To validate the notion that PtdIns(4,5)P_2 hydrolysis is dependent on candidalysin, the phosphoinositide was analyzed in TR146 cells infected with the *ece1*Δ/Δ mutant. Unlike the wild-type *C. albicans* hyphae, which caused extensive depletion of PtdIns(4,5), hyphae formed by the *ece1*Δ/Δ mutant were without effect (Figure 2G).

Candidalysin-induced membrane rupture causes $(Ca^{2+})_c$ oscillations

The preceding observations suggested that candidalysin increases the permeability of the host membrane to Ca^{2+}. That candidalysin promotes Ca^{2+} influx was previously reported using Fura-2 (Moyes et al., 2016). We confirmed and extended these observations using the genetically-encoded fluorescent Ca^{2+} sensor GCaMP6s (Chen et al., 2013). GCaMP6s has a similar affinity for Ca^{2+} as Fura-2 but has faster response kinetics and a much greater dynamic range. Representative results are presented in Figure 3A and Video S1. Candidalysin caused a rapid increase in $[Ca^{2+}]_c$, but only when Ca^{2+} was present extracellularly (Figure 3A). The responsiveness of GCaMP6s in cells bathed in Ca^{2+}-free medium was validated by adding thapsigargin (TG), an inhibitor of the SERCA pump that causes net Ca^{2+} release from the endoplasmic reticulum, with an accompanying elevation of $[Ca^{2+}]_c$ (Figure 3A). Intriguingly, in contrast to the measurements by (Moyes et al., 2016), the increase in $[Ca^{2+}]_c$ induced by candidalysin was rarely sustained; instead, multiple short-lived $[Ca^{2+}]_c$ transients were generally recorded

(C) Micrograph of PLCδ-GFP-transfected TR146 cells before (top) and after (bottom) ionomycin treatment.
(D) Immunofluorescence staining of candidalysin using the CaLF1 nanobody in invasion pockets formed by wild-type *C. albicans* (left) or by the *ece1*Δ/Δ mutant (right). Images representative of ≥30 observations in 3 independent experiments.
(E) Micrographs of PLCδ-GFP-transfected TR146 cells before (top left) and after treatment with the control peptide Ece1-VIIa (top middle), and after treatment with 30 μM candidalysin (top right) in the presence of extracellular Ca^{2+}. Bottom panels show a similar experiment in the absence of extracellular Ca^{2+}.
(F) Quantification of PM/cytosolic ratio of PtdIns(4,5)P_2 from experiments like those in (E). Data are means ± SEM of ≥3 individual experiments of each type.
(G) Micrograph of pockets induced by wild-type *C. albicans* (left) or the *ece1*Δ/Δ mutant (right). TR146 cells were transfected with the PtdIns(4,5)P_2-binding probe PLCδ-GFP. Inset in left panel shows the outline of the hypha. Images are representative of ≥ 30 cells from 3 experiments of each type. All scale bars represent 5 μm.

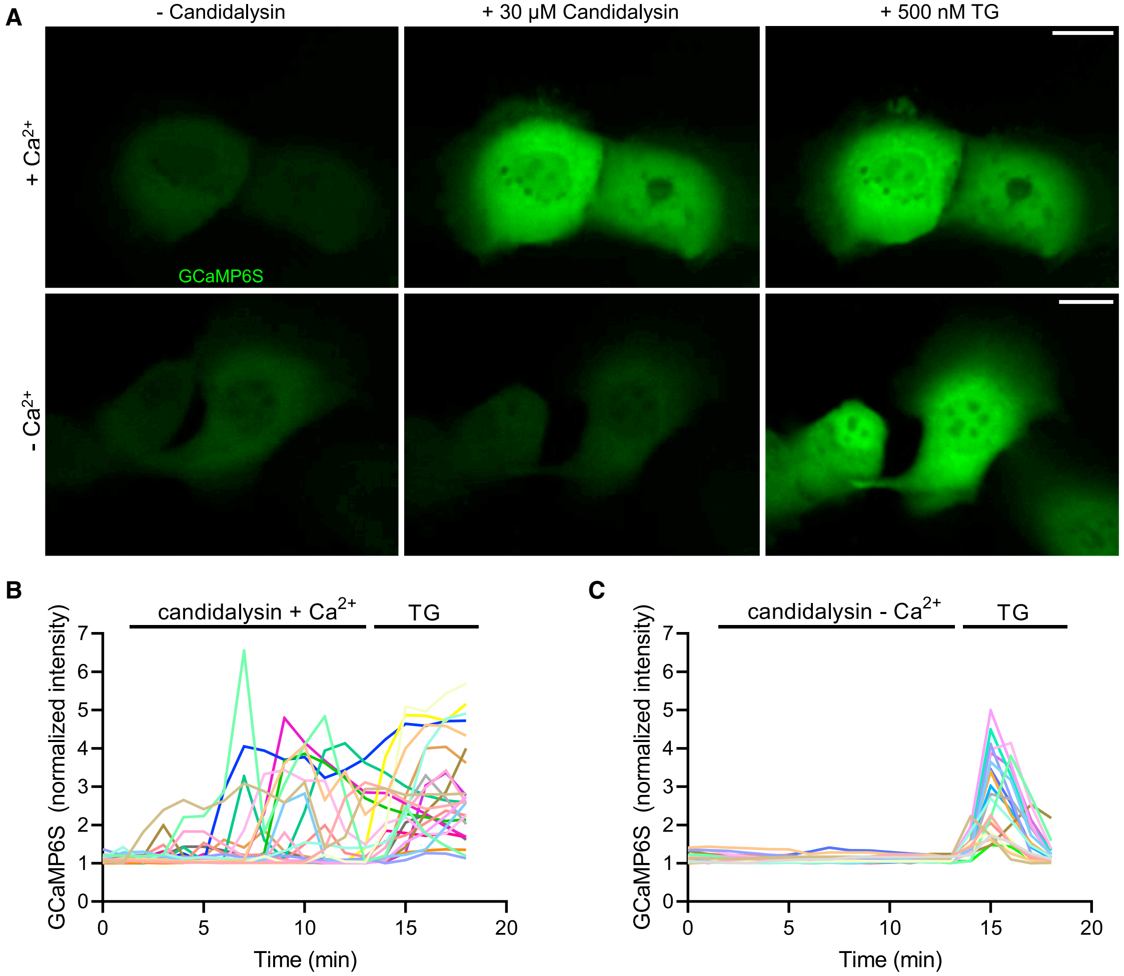

Figure 3. Candidalysin causes $[Ca^{2+}]_c$ oscillations

(A–C) TR146 cells were transfected with GCaMP6s-GFP and incubated with 30 µM candidalysin in the presence (A, top row, and B) or absence of extracellular Ca^{2+} (A, bottom row, and C). GCaMP6s fluorescence was monitored every 20 s for 12 min, followed by addition of thapsigargin (TG, 500 nM) for an additional 3 min. $[Ca^{2+}]_c$ changes, monitored using GCaMP6s, in 12 representative cells in response to candidalysin treatment in the presence of extracellular Ca^{2+}, followed by TG. (C) $[Ca^{2+}]_c$ changes of 12 representative cells after candidalysin treatment in the absence of extracellular Ca^{2+}, followed by addition of TG. Images and graphs are representative of at least 3 experiments of each type. Scale bars represent 20 µm.

(Figure 3B). Some of the cells showed up to five $[Ca^{2+}]_c$ flashes within the first 15 min after candidalysin treatment (Video S1). Of note, the epithelial cells often managed to reduce the $[Ca^{2+}]_c$ back to the basal level between or after oscillations. As shown in Figures 3B and 3C, the effect of candidalysin on GCaMP6s required the presence of extracellular Ca^{2+}. Lastly, the control peptide Ece1-VIIa did not induce $[Ca^{2+}]_c$ changes, corroborating the specificity of the changes triggered by candidalysin.

Epithelial cells respond to candidalysin by forming PM-derived blebs

The transient nature of the candidalysin-induced Ca^{2+} permeability changes was suggestive of intervening inactivation or extrusion of the pore-forming toxin. To explore this possibility, we visualized the PM during the course of exposure to candidalysin using the lipophilic fluorescent dye FM4-64, which is sufficiently small to enter *C. albicans*-containing invasion pockets that otherwise restrict the entry of larger molecules such as ConA (Figure 4A). Strikingly, the dye revealed a marked convolution of the host membrane in the distal regions of the pocket, where candidalysin is most often detected, with formation of multiple blebs that were trapped between the hypha and the epithelial cell (Figure 4A). Membrane blebbing was only prominent in long invasion pockets, which take 4–6 h to form, while short pockets remained intact. Interestingly, the fluorescence intensity of FM4-64 in blebs was significantly higher than in regions of the membrane that were intact, suggesting that the dye—which does not permeate intact bilayers—reaches both sides of the bleb membrane; we interpret the increased fluorescence as indicative of rupture of the bleb membrane. That candidalysin is responsible for formation of the blebs was suggested by

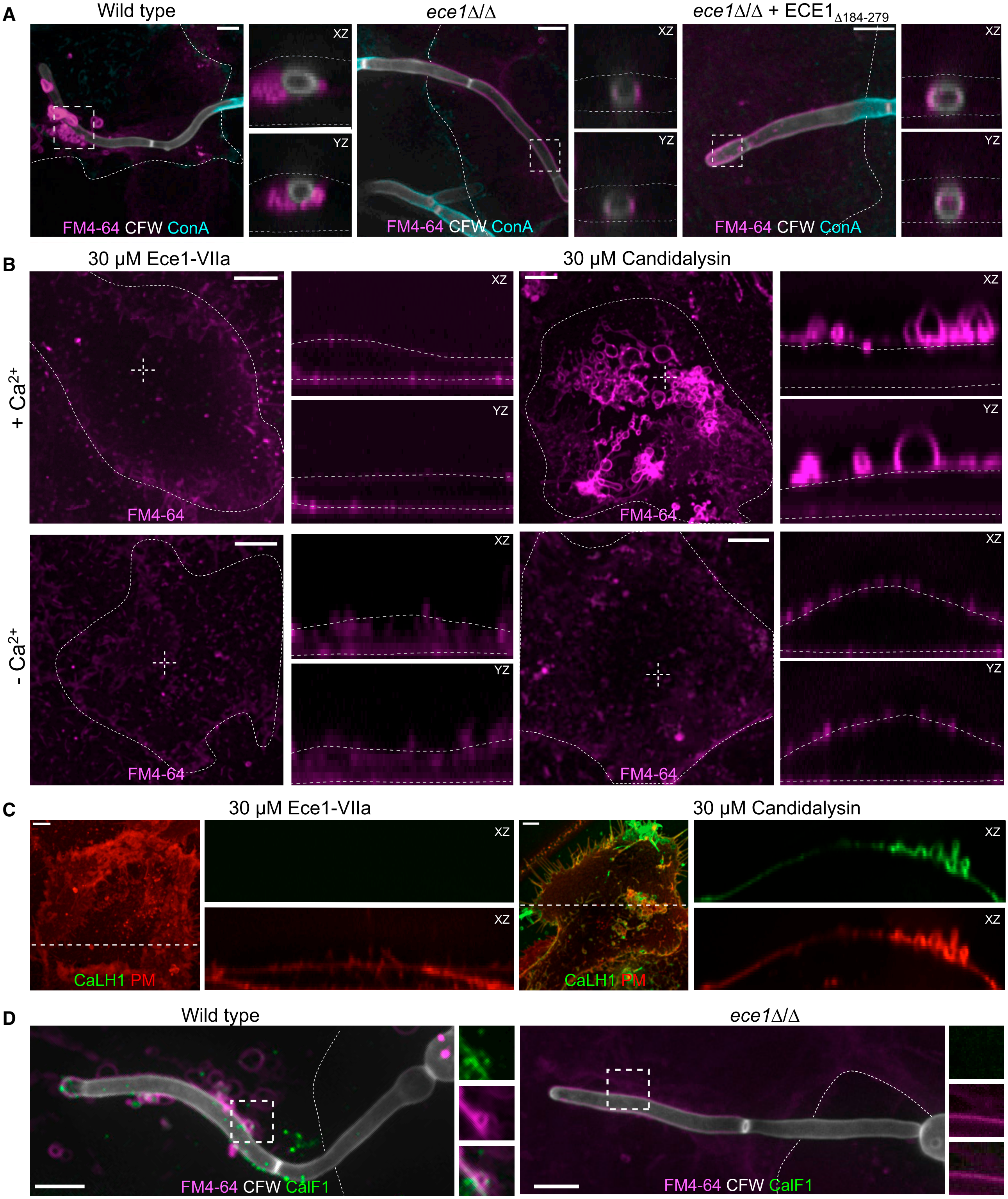

Figure 4. Epithelial cells respond to rupture by the formation of PM-derived blebs

(A) Representative micrographs of TR146 invasion pockets induced by wild-type, *ece1*Δ/Δ, and *ece1*Δ/Δ+*ECE1*Δ184-279 strains of *C. albicans*. PM bleb formation was assessed by staining non-permeabilized cells with 10 μM FM4-64. Side panels show the XZ and YZ slices corresponding to areas marked by dashed boxes.

(legend continued on next page)

CellPress
OPEN ACCESS

analysis of comparable long pockets generated by *ece1*Δ/Δ hyphae (Figure 4A). In these instances, labeling with FM4-64 showed the membrane lining the pocket to be continuous, with no evidence of blebs or discontinuities. Similarly, we found no evidence of damage or bleb formation in pockets formed by *ece1*Δ/Δ + *ECE1*$_{\Delta 184-279}$ hyphae, which express all Ece1-derived peptides except candidalysin (Figure 4A).

PM blebs were also formed when synthetic candidalysin was added to TR146 cells (Figure 4B). In this case, bleb formation was visible as early as 10 min after addition of the toxin and coincided with the $[Ca^{2+}]_c$ oscillations (Video S2). Candidalysin induced PM blebbing only when Ca^{2+} was present in the medium (Figure 4B), which suggests that the blebs form in response to the previously documented Ca^{2+} influx. Accordingly, no PM damage or bleb formation was visible when the cells were incubated with peptide Ece1-VIIa (Figure 4B), which similarly failed to induce Ca^{2+} influx.

Next, we investigated whether candidalysin-induced PM blebs contained candidalysin by staining the synthetic toxin with the α-candidalysin nanobodies. The labeled CaLH1 nanobody localized to PM blebs induced by synthetic candidalysin, but it did not stain cells incubated with peptide Ece1-VIIa (Figure 4C). Candidalysin-positive blebs could be visualized apically within 10 min of candidalysin exposure (Figure S1A). At later time points, the blebs pinched off and separated from the cells, removing PM material and candidalysin from the epithelial surface in the process (Figure S1B). The PM blebs did not retract back into the cell body (Figure S1C; Video S3). Moreover, bleb staining remained higher than that of the PM, indicating discontinuity between the bleb and the PM. Since FM4-64 diffuses laterally along the membrane, the observed diffusion barrier likely reflects separation of the bleb from the PM.

The CaLF1 nanobody detected secreted candidalysin in the blebs of wild-type *C. albicans* invasion pockets, but not in those of the *ece1*Δ/Δ strain (Figure 4D). We also investigated whether invasion pockets generated by other *C. albicans* clinical isolates developed PM-derived blebs similar to the ones formed by the SC5314 strain used throughout this study. We found that induction of PM blebs by the clinical strains correlated with their ability to secrete candidalysin. *C. albicans* isolates HUN96 and 101 secreted detectable levels of candidalysin and induced PM blebs (Figure S1D). In contrast, we failed to detect candidalysin and PM blebs in pockets formed by isolate 529L (Figure S1D); this strain expresses a variant candidalysin that is secreted at a reduced rate (Liu et al., 2021). We attribute the failure to form blebs to the reduced secretion, rather than to structural differences between the candidalysins, because *ab initio* structure predictions for the candidalysin peptides secreted by SC5314 and 529L showed only minor differences that do not markedly affect their hydrophobicity, charge, or polarity (Figures S1E vs. S1F). Accordingly, the calcium influx, cytokine secretion, and ultimate cell damage effected by treatment with synthetic 529L and SC5314 candidalysins were indistinguishable (J.N., unpublished). We therefore propose that entry of Ca^{2+} via candidalysin triggers formation of PM-derived blebs that contain the toxin. These blebs are trapped inside the invasion pocket but can be released into the culture medium when induced by synthetic candidalysin. Shedding of the toxin along with the blebbing membrane terminate the influx of Ca^{2+}, at least temporarily, accounting for the transient nature of the oscillations detected by GCaMP6s.

Permeabilization of the PM is followed by recruitment of ALG-2 and ESCRT, leading to shedding of blebs and PM repair

Most cells have the ability to repair their PM upon rupture, a process that restores internal homeostasis and prevents cell death. Repair responses are often mediated by the influx of extracellular Ca^{2+}. One such repair mechanism is the ALG-2-dependent recruitment of the ESCRT-III complex. ALG-2 has two high-affinity and one low-affinity Ca^{2+}-binding sites; upon binding Ca^{2+}, ALG-2 undergoes conformational changes that enable it to associate with the membrane (Jia et al., 2001). As it accumulates at sites of membrane rupture, where Ca^{2+} influx occurs, ALG-2 recruits ALG-2-interacting protein X (ALIX) and CHMP proteins of the ESCRT-III complex (Jimenez et al., 2014; Scheffer et al., 2014). Assembly of ESCRT-III components constricts and extrudes the damaged membrane. The ensuing scission of the damaged patch results in repair and shedding of the agent that caused the membrane damage. We tested whether this machinery was involved in the blebbing and in the termination of the permeability enhancement caused by candidalysin. To this end, the epithelial cells were transfected with fluorescent chimeras of CHMP2a, CHMP4B, and CHMP6. As shown in Figures 5A and S2, these ESCRT-III components were recruited to invasion pockets containing wild-type *C. albicans*. Furthermore, CHMP2a accumulated in PM blebs stained by FM4-64 inside long invasion pockets containing wild-type *C. albicans* (Figure 5B), but not in invasion pockets formed by the *ece1*Δ/Δ hyphae (Figure 5B). Similarly, CHMP2a was recruited to the base of blebs formed by TR146 cells treated with synthetic candidalysin; no such recruitment was observed when cells were exposed to Ece1-VIIa (Figure 5C).

To confirm the Ca^{2+}-dependence of the recruitment of the ESCRT-III machinery, *C. albicans*-induced invasion pockets were stained for ALG-2. Like the ESCRT-III components

(B) Micrographs of TR146 cells treated with 30 μM Ece1-VIIa peptide or candidalysin in the presence or absence of extracellular Ca^{2+}. PM bleb formation was assessed by staining non-permeabilized cells with 10 μM FM4-64. Outlines of TR146 cells indicated by dotted lines. Side panels show the XZ and YZ slices corresponding to the areas marked by the white dashed crosshairs.

(C) Micrographs of TR146 cells transfected with PM-RFP and treated with either 30 μM Ece1-VIIa peptide or candidalysin. Localization of candidalysin was assessed by staining non-permeabilized cells with CaLH1 nanobody. Side panels show XZ slices of individual channels, corresponding to the dashed white lines. Outlines of cells are indicated by dotted lines.

(D) Representative confocal micrographs of TR146 invasion pockets induced by wild-type and *ece1*Δ/Δ strains of *C. albicans*. Bleb formation and candidalysin were visualized by staining non-permeabilized cells with 10 μM FM4-64 and CaLF1 nanobody, respectively. Side panels are XY slices showing CalF1 (green), FM4-64 (magenta), and merged channels, for areas marked by dotted boxes. Scale bars represent 5 μm. Images representative of ≥3 experiments of each type. See also Figure S1.

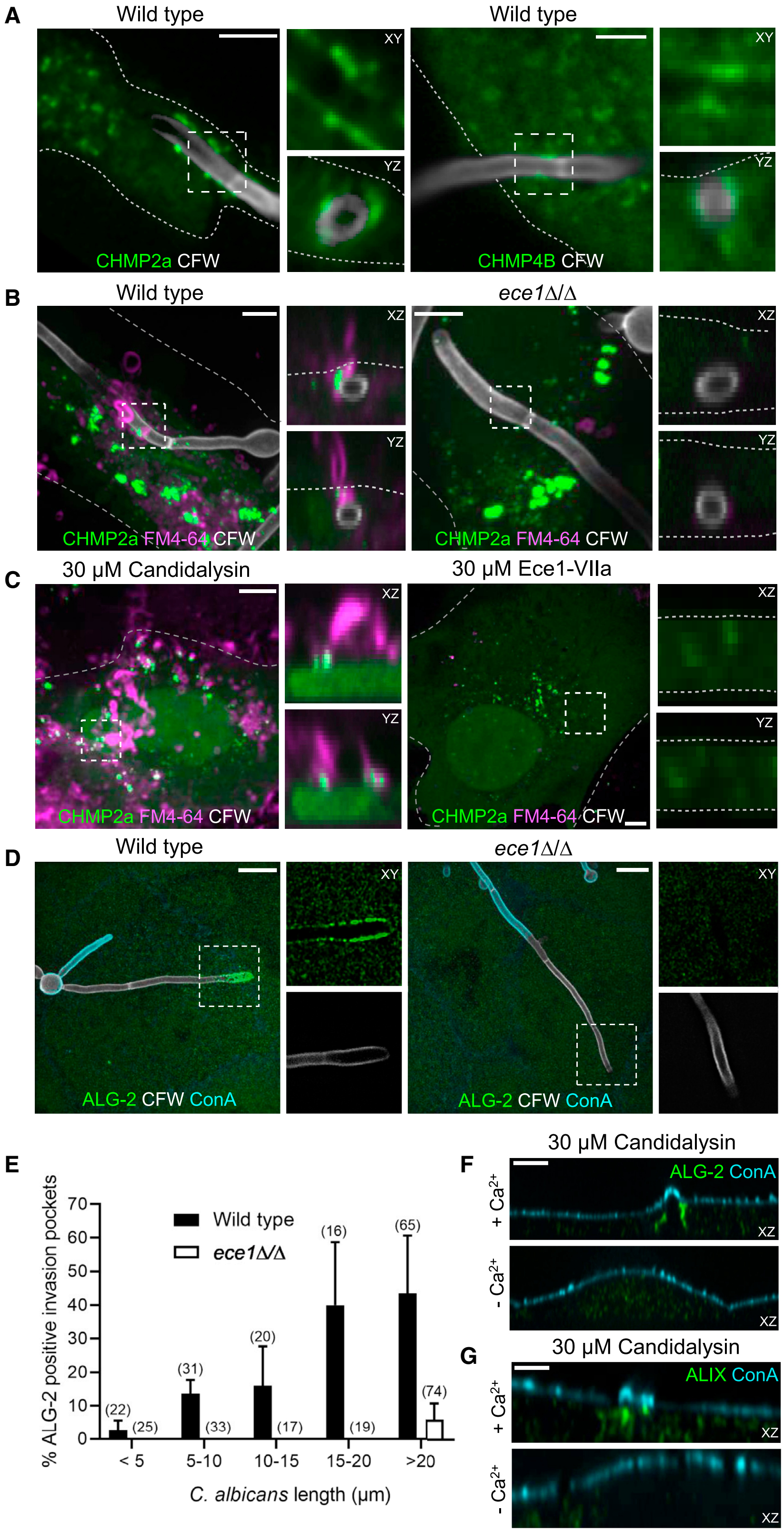

Figure 5. PM damage is followed by recruitment of ALG-2 and ESCRT-III leading to PM repair and shedding of the damaged membrane

(A) Micrograph of invasion pockets containing *C. albicans*. TR146 cells were transfected with CHMP2a-GFP (left), CHMP4B-mCherry (right). Side panels show the XY and YZ slices corresponding to areas marked by the dotted boxes.

(B) Micrograph of pockets containing wild-type or *ece1*Δ/Δ *C. albicans*. TR146 cells were transfected with CHMP2a-GFP (left), CHMP4B-mCherry (right), and PM bleb formation was assessed staining non-permeabilized cells with 10 μM FM4-64. Side panels show the XZ and YZ slices corresponding to the areas marked by the dotted boxes.

(C) Micrographs of TR146 cells treated with 30 μM Ece1-VIIa peptide or candidalysin. Cells were transfected with CHMP2a-GFP and PM bleb formation assessed by staining non-permeabilized cells with 10 μM FM4-64. (A–C) Outlines of TR146 cells indicated by dotted lines. Scale bars represent 5 μm.

(D) Confocal micrographs of TR146 cells infected with wild-type (left panel) or *ece1*Δ/Δ mutant *C. albicans* (right panel). ALG-2 was detected by immunofluorescence. Scale bar represents 10 μm.

(E) Quantification of ALG-2 accumulation as a function of invasion pocket length, from experiments like those in (D). Data are means ± SEM of ≥3 individual experiments.

(F and G) Micrographs of TR146 cells treated with 30 μM candidalysin. ALG-2 (F) and ALIX (G) detected by immunofluorescence and PM blebs detected staining the PM with ConA. Images are representative of ≥3 experiments of each type. See also Figure S2.

CellPress
OPEN ACCESS

CHMP2a/4B/6, ALG-2 accumulated in invasion pockets formed by wild-type *C. albicans* hyphae, but not those induced by the *ece1*Δ/Δ mutant (Figure 5D). ALG-2 accumulation was dependent on the length of the invasion pocket, increasing with the depth of the invagination (Figure 5E). Additionally, ALG-2 as well as ALIX accumulated under nascent PM blebs induced by synthetic candidalysin, in a Ca^{2+}-dependent manner (Figures 5F and 5G). Our data suggest that the observed blebs inside invasion pockets and at the surface of candidalysin-treated TR146 cells are indicative of a repair response triggered by Ca^{2+} influx that leads to the sequential recruitment of ALG-2, ALIX, and ESCRT-III.

Silencing ALG-2 prevents ESCRT-III-mediated membrane repair and curtails epithelial viability

We hypothesized that recruitment of the ESCRT-III machinery to sites of invasion was essential to maintain epithelial viability during infection. This premise was tested by assessing the effects of candidalysin on the survival of cells where ALG-2 had been depleted. Using siRNA, ALG-2 was consistently depleted by ≈70% (Figures 6A and 6B). Synthetic candidalysin induced markedly fewer PM blebs in ALG-2-silenced cells than in the paired controls (Figures 6C and 6D). Of note, the $[Ca^{2+}]_c$ accumulation induced by candidalysin was greater in ALG-2-silenced cells (Figure 6E), consistent with the notion that scission of blebs containing the toxin antagonizes its permeabilizing effects, enabling transient restoration of near-normal $[Ca^{2+}]_c$. Indeed, in silenced cells the candidalysin-induced oscillatory transients seen in otherwise untreated cells were replaced by greater and more prolonged increases in $[Ca^{2+}]_c$ (Figure 6F).

Upon treatment with candidalysin, the mitochondrial membrane potential (measured as accumulation of rhodamine 123), which is preserved in cells treated with control siRNA, was largely dissipated in cells depleted of ALG-2 (insets, Figure 6C). The latter observation is suggestive of cell morbidity or death in the depleted cells. Two conventional approaches were applied to more specifically assess cell viability: staining with the nominally impermeant dyes DAPI and propidium iodide. As shown in Figure 6C, ALG-2-depleted cells displayed increased staining with DAPI. A systematic study revealed that cell death was significantly greater in cells where ALG-2 had been silenced (Figures 6H–6G). Transient overexpression of CHMP4B did not protect TR146 cells from the cytotoxic effects of candidalysin, suggesting that overexpression of a single component of the ESCRT machinery is insufficient to protect the cells against the toxin (Figures S3A and S3B). These findings suggest that ALG-2/ESCRT-III-mediated membrane repair is required for the cells to tolerate sublethal concentrations of candidalysin.

We also tested the effects of IL-22, which was reported to protect epithelia during oral candidiasis *in vivo* (Conti et al., 2009; Bichele et al., 2018; Aggor et al., 2020). Pre-incubation with recombinant IL-22 for 48 h did not protect TR146 cells against candidalysin-induced cell damage (Figure S3C).

Lysosome exocytosis repairs mechanical damage caused by very long *C. albicans* hyphae

Mechanisms other than ESCRT-III-mediated membrane scission can also affect membrane repair. These include the exocytosis of lysosomes, a process that is similarly triggered by elevated $[Ca^{2+}]_c$. Because candidalysin mediates Ca^{2+} influx, we considered whether lysosomal fusion is also promoted by the toxin. Addition of synthetic candidalysin to the TR146 cells induced lysosome exocytosis when extracellular Ca^{2+} was present. As depicted in Figure 7A, upon treatment with candidalysin an epitope on LAMP-1 normally facing the lumen of lysosomes becomes exposed on the membrane surface and is detectable in non-permeabilized cells, indicating exocytic insertion into the PM. The exocytosis of lysosomes triggered by candidalysin was often observed within the first hour of co-incubation with *C. albicans*, but it became statistically significant only after 2 h (Figure 7B). A comparable treatment with the control Ece1-VIIa peptide did not induce lysosome secretion (Figure 7B).

Unexpectedly, LAMP-1 staining was also observed in a fraction of the cells in the absence of extracellular Ca^{2+}. However, close examination by serial confocal sections of cells exposed to candidalysin in Ca^{2+}-free medium revealed that the PM was not labeled (Figure 7A), ruling out the occurrence of exocytosis. Instead, endomembrane structures were immunolabelled with the LAMP-1 antibody. We concluded that, in the absence of Ca^{2+}, the monovalent cation fluxes catalyzed by candidalysin alter the ionic balance of endomembrane organelles, including lysosomes, causing their lysis and exposing the luminal aspect of LAMP-1 to the antibody. The ionic imbalance is also likely to account for the loss of mitochondrial potential (Figure 6C).

Because ESCRT-III-mediated PM repair mitigated the increases in $[Ca^{2+}]_c$ prompted by candidalysin, we predicted that the secretion of lysosomes would be enhanced in its absence. This was tested by silencing ALG-2 prior to challenging the cells with the toxin. Consistent with the requirement for elevated $[Ca^{2+}]$, candidalysin-induced lysosome exocytosis was increased moderately when ALG-2 was depleted, although the change did not reach statistical significance ($p = 0.16$; Figure 7C). We suggest that ESCRT-III-mediated membrane blebbing and lysosome exocytosis serve complementary roles to mitigate candidalysin-induced PM damage.

As mentioned earlier, LAMP-1 was detectable in long invasion pockets. We assumed that localized secretion of candidalysin was responsible for this occurrence. Surprisingly, distinct insertion of LAMP-1 into the lining of the long invasion pockets was also noted when cells were infected with *ece1*Δ/Δ mutant hyphae (Figures 7D and 7E), suggesting that extreme mechanical strain can promote Ca^{2+} entry and lysosome exocytosis even in the absence of candidalysin. Mechanosensitive Ca^{2+} channels or transient membrane rupture could enable Ca^{2+} influx when membrane invagination is extreme. Accordingly, LAMP-1 was not found in invasion pockets shorter than 20 μm (Figure 7D), suggesting that lysosome exocytosis is a late, secondary response to PM damage.

Two other features are noteworthy. First, plasmalemmal LAMP-1 was generally detected in regions where F-actin had been lost, likely as a consequence of Ca^{2+} entry. Secondly, despite the insertion into the membrane lining the invasion pocket of lysosomes, which are rich in proton-pumping V-ATPases, the fluid within the invagination did not become detectably acidic (Figure S4), in all likelihood because H^+ was able to diffuse readily out of the invasion pocket.

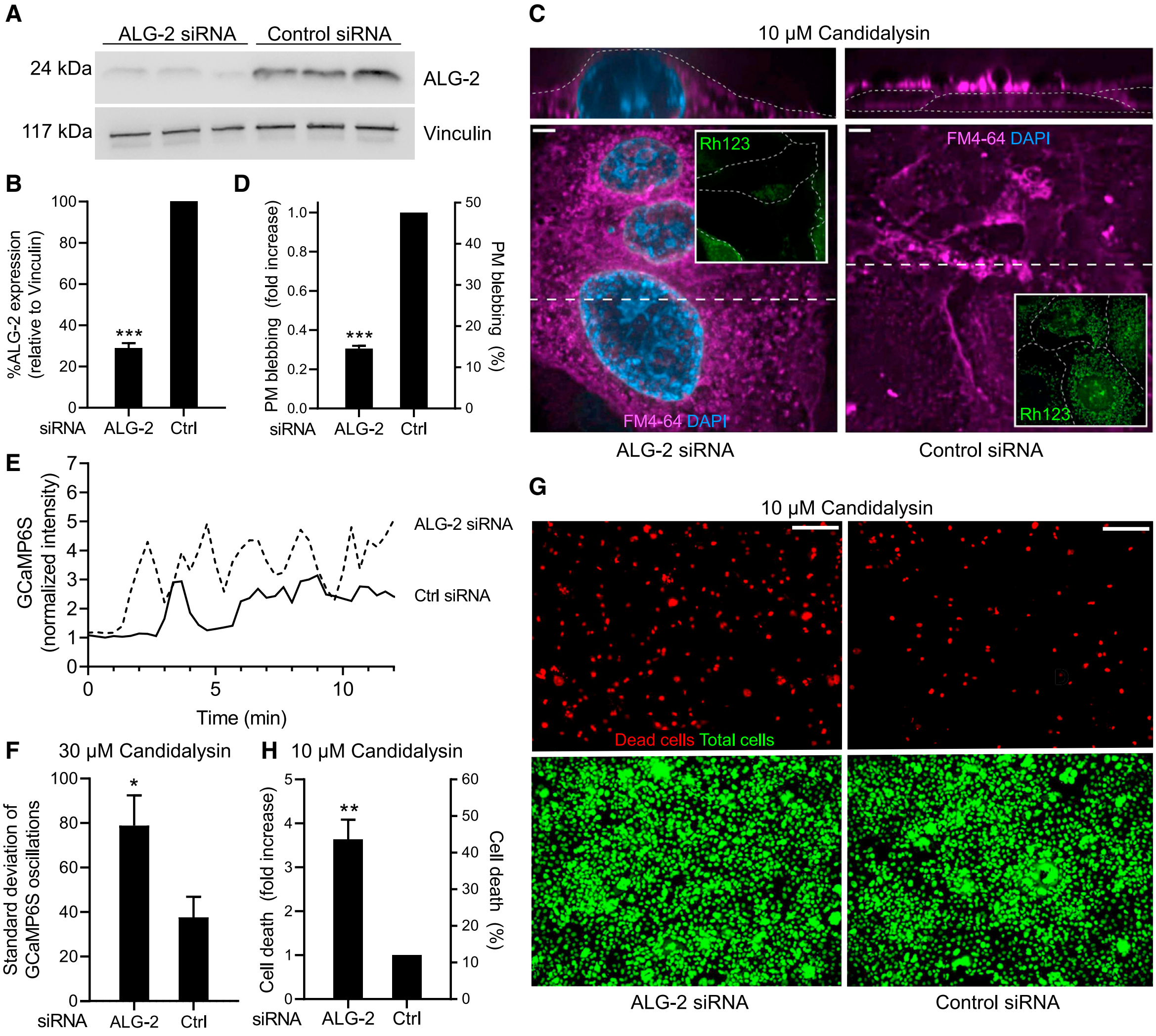

Figure 6. Silencing ALG-2 prevents ESCRT-III-mediated membrane repair and curtails epithelial viability

TR146 cells were treated with ALG-2 siRNA or scrambled siRNA (control).

(A and B) ALG-2 silencing verified by immunoblotting using vinculin as loading control. Data in B are means ± SEM of 6 separate determinations.

(C) Representative micrographs of TR146 cells treated with 30 μM candidalysin. Membranes were stained with FM4-64 and PM rupture was detected by endomembrane staining and using DAPI. Insets: mitochondrial membrane potential assessed with rhodamine-123. Outlines of TR146 cells indicated by dotted lines. Scale bar represents 5 μm; dashed line indicates the position where the *XZ* image on top was constructed.

(D) Quantification of PM blebbing from experiments like those in C. Data are means ± SEM of ≥3 individual experiments.

(E) Representative time courses of $[Ca^{2+}]_c$ determinations made using GCaMP6s following 30 μM candidalysin treatment in Ca^{2+}-containing medium. TG (500 nM) was added where indicated.

(F) Estimation of the $[Ca^{2+}]_c$ changes induced by 10 μM candidalysin. Standard deviation of the $[Ca^{2+}]_c$ changes induced by 10 μM candidalysin in cells treated with control or ALG-2 siRNA. Data are means ± SEM of at ≥3 individual experiments.

(G) Representative micrographs of TR146 cells treated with 10 μM candidalysin and stained with propidium iodide to identify dead cells, then permeabilized with 0.2% Triton X-100 and counter-stained to visualize all nuclei using Sytox Green. Scale bar represents 200 μm.

(H) Quantification of cell death from experiments like those in (C). Data are means ± SEM of ≥3 individual experiments. See also Figure S3.

DISCUSSION

We investigated the manner by which *C. albicans* invades epithelial cells and the mechanisms whereby the epithelium contends with the stresses imposed by growing hyphae. Our studies indicate that, unlike the frustrated phagosome that forms when macrophages confront *C. albicans* hyphae, the invagination that the fungus forces when growing on epithelial cells is largely of plasmalemmal origin. The frustrated phagosome is separated from the bulk of the PM by a sturdy actin cuff that forms a diffusional barrier that contains endomembrane proteins and lipids within the invagination. However, in the epithelial pocket, the specialized actin cuff is missing, and in fact, discontinuities in the submembranous cortical actin appear progressively as the hyphae burrow deeper into the cell. The detachment of cortical actin is secondary to the loss of plasmalemmal PtdIns(4,5)P_2,

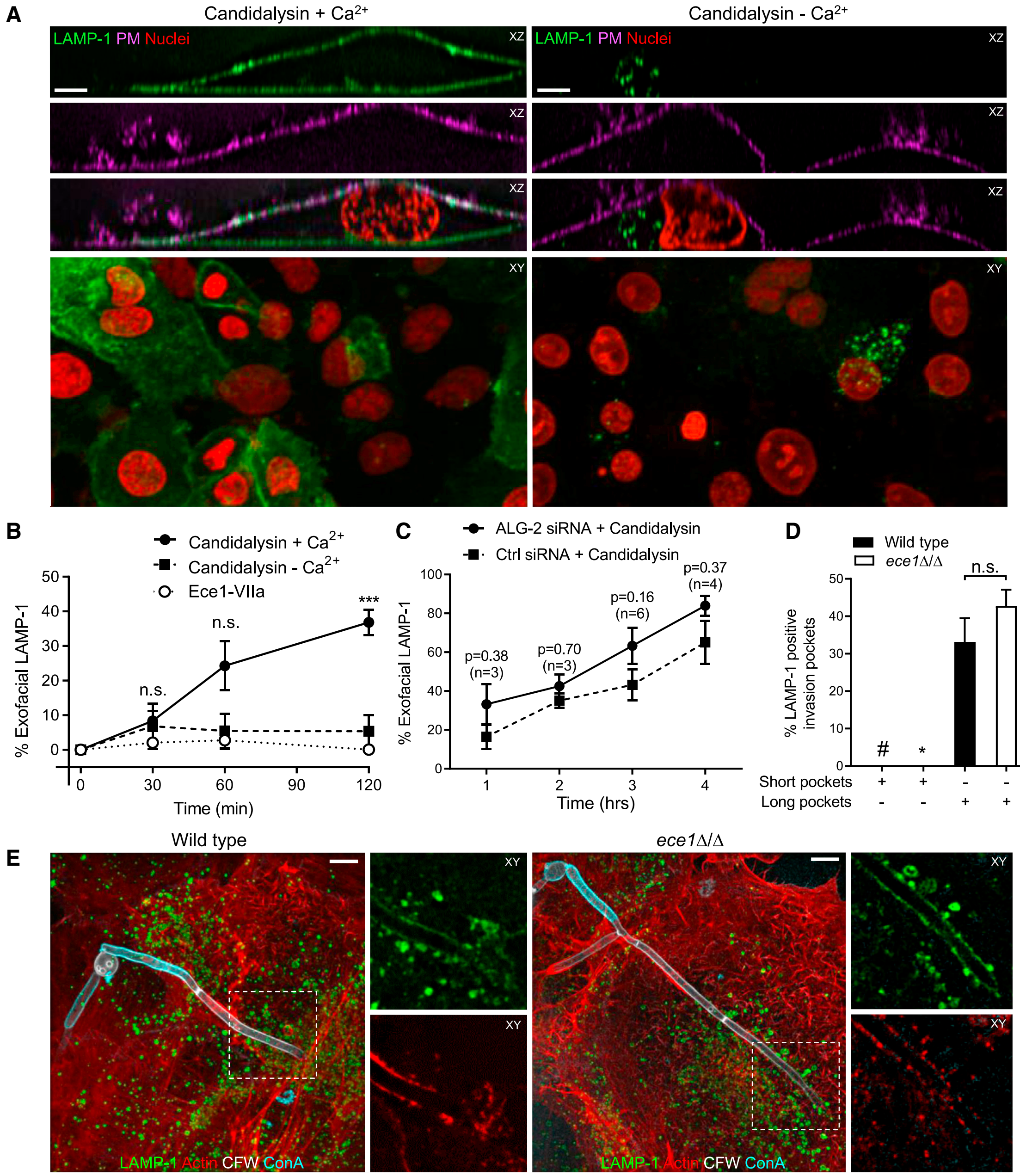

Figure 7. Lysosome exocytosis contributes to repair PM damage during *C. albicans* infection

(A) Representative micrographs of TR146 cells treated with 30 μM candidalysin in the presence or absence of extracellular Ca^{2+}. Lysosome exocytosis assessed detecting exofacial LAMP-1 staining in non-permeabilized cells with an antibody directed to a luminal epitope. The PM was stained with ConA and nuclei were stained with propidium iodide (PI) to visualize all cells. Scale bars represent 5 μm in *XZ* panels and 15 μm in *XY* panels.

(B) Quantification of exofacial LAMP1 from experiments like (A). Data are means ± SEM of ≥3 individual experiments of each type.

(C) Quantification of exofacial LAMP-1 in TR146 cells treated with ALG-2 siRNA or control siRNA. Data are means ± SEM. p value and experimental number of determinations (n) shown above each data point.

(D) Quantification of LAMP-1 insertion in invasion pockets formed in TR146 cells by wild-type or *ece1*Δ/Δ mutant *C. albicans*. Data are means ± SEM of ≥3 individual experiments. #, LAMP-1 not detected in >50 short pockets containing wild-type *C. albicans*. *, LAMP-1 not detected in >50 short pockets containing *ece1*Δ/Δ *C. albicans*.

(E) Representative micrographs of cells infected by wild-type (left panel) or *ece1*Δ/Δ mutant *C. albicans* (right panel). Scale bar represents 5 μm. See also Figure S4.

which is in turn caused by influx of extracellular Ca^{2+} through candidalysin. This notion is supported by the finding that the candidalysin-deficient *ece1*Δ/Δ strain had no discernible effect on PtdIns(4,5)P_2 (Figure 2G) or on cortical actin (Figure 2D). That only long pockets show loss of membrane integrity suggests that candidalysin—which starts being secreted when the yeast transition to hypha—needs to accumulate over time to reach concentrations that effect permeabilization. Accordingly, the loss of PtdIns(4,5)P_2 and the associated actin depolymerization could be replicated by addition of synthetic candidalysin to otherwise untreated cells, implying that the toxin suffices to produce the alterations observed in infected cells. Another peptide encoded by the *ECE1* gene, peptide VIIa, was without effect, validating the specificity of candidalysin (Figure 2D).

Two lines of evidence support the conclusion that Ca^{2+} influx through candidalysin triggers the changes in PM structure. First, the loss of PtdIns(4,5)P_2 and F-actin was only observed in the presence of extracellular Ca^{2+}, failing to occur when the cation was omitted. Second, $[Ca^{2+}]_c$ was found to increase when cells were treated with candidalysin, but not with the Ece1-VIIa peptide, and only when external Ca^{2+} was present. Interestingly, the $[Ca^{2+}]_c$ increases were generally transient, followed by restoration of the basal levels, which suggests that the pores formed by candidalysin were short-lived. This may reflect inherent instability of the multimeric pore formed by candidalysin, but it is most likely an indication of membrane repair events that extrude or inactivate the toxin. In support of this, oscillatory recruitment of membrane repair machinery has been observed in cells treated with the bacterial pore-forming toxin streptolysin (SLO) (Babiychuk et al., 2009; Keyel et al., 2011). Similarly, we found that $[Ca^{2+}]_c$ oscillations in response to candidalysin became more pronounced and sustained when repair mechanisms were overwhelmed or impaired. As with SLO, the host membrane repair mechanisms culminate in the release of blebs from the host PM, which contained candidalysin (Figures 4, S1 and Video S3). Of note, bleb release has also been observed for two short toxin peptides that are structurally similar to candidalysin: melittin (Černe et al., 2013) and gomensin (Paredes-Gamero et al., 2012).

As a commensal and opportunistic pathogen, *C. albicans* continuously interacts with the host. We found that the host implements robust responses to offset the stresses *C. albicans* creates on the epithelial PM during invasion. By electron microscopy, the invasion pocket was reported recently to have *inflated membranes* with numerous associated vesicles (Lechat, 2021), reminiscent of the blebs we document. Our cell-based studies are also in accordance with *in vivo* mouse and zebrafish candidiasis models that link *ECE1* expression with *C. albicans* pathogenicity and invasiveness (Moyes et al., 2010; Swidergall et al., 2019). *C. albicans* clinical isolates have been studied *in vivo* and characterized as host-invading or colonizing (Schönherr et al., 2017; Kirchner et al., 2019). Interestingly, we found that *C. albicans* isolates that secreted detectable amounts of candidalysin, regardless of invading or colonizing capability *in vivo*, induced blebs in invasion pockets (Figure S1D). One isolate tested, 529L, formed hyphae but did not secrete detectable candidalysin nor did it induce membrane blebs. Notably, the 529L isolate encodes a variant of Ece1, which is processed less efficiently (Liu et al., 2021). Consequently, less mature candidalysin is delivered into the invasion pocket (Figure S1F), and damage levels caused by invading 529L hyphae remain low.

As the ability to produce hyphae has been conserved in most clinical isolates, hyphal formation likely confers an evolutionary advantage to *C. albicans*, including during its commensal phase (Witchley et al., 2019). Therefore hyphal formation per se would be insufficient to cause damage. Only an increased burden of invasive hyphae, which deliver a critical level of candidalysin into the invasion pocket (Mogavero et al., 2021), causes epithelial damage followed by necrotic cell death (Ho et al., 2019; Blagojevic et al., 2021). Indeed, our work demonstrates that epithelial repair is an additional major player in this protective mechanism. Given that epithelial cells must tolerate moderate levels of invading hyphae in a commensal setting, only an overwhelming level of candidalysin delivered into the invasion pocket by invading hyphae, which cannot be compensated by epithelial repair, would be associated with epithelial damage, necrosis, and disease.

The key finding of this study is that epithelial cells implement two different types of repair responses to counteract the actions of *C. albicans* hyphae. These responses not only protect the host from fungal dissemination and detrimental infection, but maintain the homeostatic balance with commensal *C. albicans* to preserve the ostensibly beneficial relationship. The protective responses consist of PM blebbing that disposes of damaged membranes along with the permeabilizing candidalysin, and exocytosis of endomembranes that patch the ruptured surface. Both of these responses are triggered by elevation of $[Ca^{2+}]_c$, which is also a mediator of the cellular damage. While seemingly paradoxical, these findings imply that the epithelial cells are finely tuned to sense and counteract the very parameter that effects the damage. ALG-2 and ESCRT-III are the first responders. They accumulate in sites of hyphal infection and at the base of the blebs induced by synthetic candidalysin. The toxin is in fact detected in such blebs, confirming that they serve to eliminate it from the cell surface. While ESCRT-III repair has been characterized in cells subjected to bacterial or chemical damage (Jimenez et al., 2014; Scheffer et al., 2014), it had not previously been reported during interaction with fungal cells. When triggered by candidalysin, the ESCRT-III system protects the host cell; this is indicated by the reduced viability of epithelial cells that had been depleted of ALG-2 prior to treatment with the toxin (Figures 6E and 6F). It is noteworthy that ESCRT-generated vesicles are generally small (100–200 nm), below the limit of resolution of conventional light microscopy, whereas at least a fraction of the vesicles generated by candidalysin were significantly larger. This observation, however, is not unprecedented, since similar vesicles–ranging from 200 nm to 2 μm—were also reported to be extruded by cells treated with SLO, which also triggers an ESCRT-dependent process (Keyel et al., 2011) and in cells exposed to pneumolysin (Wolfmeier et al., 2016).

Unlike the recruitment of the ESCRT-III complex, lysosome exocytosis occurred both in the wild-type and the *ece1*Δ/Δ mutant. We therefore concluded that the invasion pocket can be damaged by means other than candidalysin. *C. albicans* secretes different hydrolytic enzymes that could conceivably cause

membrane damage (Niewerth and Korting, 2001; Schaller et al., 2005; Westman et al., 2019). Alternatively, the damage may be the consequence of mechanical strain exerted by the growing hyphae, which can attach firmly to host receptor proteins. Indeed, mechanically induced rupture has been shown to occur during *C. albicans* filamentation inside macrophages, leading to phagosomal alkalinization and expansion (Westman et al., 2018, 2020). That long invasion pockets showed evidence of lysosome fusion suggests that other lysosomal components must have been delivered to the invasion pocket, including lysosomal hydrolases and the vacuolar H^+-ATPase. However, we were unable to detect localized acidification within the invasion pocket using acidotropic dyes (Figure S4), in all likelihood because H^+ can readily diffuse outward, inasmuch as significantly larger molecules—such as the nanobodies used for candidalysin detection—access the invagination.

In summary, we describe that two types of injuries—one mediated by the pore-forming toxin candidalysin, the other by mechanical stress—result from the growth of *C. albicans* hyphae on epithelial cells. To minimize the ensuing damage, host cells activate two distinct repair mechanisms that counteract the damage minimizing epithelial damage, inflammation and transepithelial dissemination. In this manner, epithelia can maintain a homeostatic balance that enables the commensal relationship with the most common opportunistic fungal pathogen, *C. albicans*.

Limitations of the study

That the effects of candidalysin on mitochondrial potential are caused indirectly by changes in cytosolic ion concentrations remains speculative and needs to be validated directly. Similarly, the proposed dependence of actin dissociation on PtdIns(4,5)P_2 hydrolysis must be confirmed experimentally. Lastly, additional studies are needed to validate the impact of the repair mechanisms *in vivo*.

STAR★METHODS

Detailed methods are provided in the online version of this paper and include the following:

- KEY RESOURCES TABLE
- RESOURCE AVAILABILITY
  - Lead contact
  - Materials availability
  - Data and code availability
- EXPERIMENTAL MODEL AND SUBJECT DETAILS
  - *Candida albicans* strains and cultivation
  - Cell lines
- METHOD DETAILS
  - Plasmids and reagents
  - DNA transfection
  - Epithelial invasion assay
  - Cytosolic Ca^{2+} measurements using pGP-CMV-GCaMP6s
  - Visualization of TR146 membrane blebbing in response to candidalysin
  - ALG-2 immunostaining
  - siRNA-mediated silencing and immunoblotting of ALG-2
  - Cell damage assays using siRNA-treated TR146 cells
  - LAMP-1 immunostaining of invasion pockets
  - Exofacial LAMP-1 immunostaining
  - Visualization of TR146 membrane debris and membrane blebs
  - Candidalysin peptide structure prediction and modeling
  - Immunoblotting of CHMP4B and calculations for cell viability experiments
  - Effect of IL-22 on viability of TR146 cells
  - Measuring the acidity of *C. albicans*-containing invasion pockets
  - Microscopy
- QUANTIFICATION AND STATISTICAL ANALYSIS
  - Image analysis and statistics

SUPPLEMENTAL INFORMATION

Supplemental information can be found online at https://doi.org/10.1016/j.celrep.2021.110187.

ACKNOWLEDGMENTS

J.W. was supported by the Swedish Society of Medicine, the Foundation Blanceflor Boncompagni Ludovisi, née Bildt, and Restracomp. B.H. is supported by the German Research Foundation (Deutsche Forschungsgemeinschaft, DFG) Priority Programme 2225 "Exit strategies of intracellular pathogens" and within the Cluster of Excellence "Balance of the Microverse", under Germany's Excellence Strategy – EXC 2051 – Project-ID 390713860. J.R.N is supported by Wellcome Trust (214229_Z_18_Z), National Institutes of Health (DE022550), and the NIH Research at Guys and St. Thomas's NHS Foundation Trust and the King's College London Biomedical Research Centre (IS-BRC-1215-20006). S.G. is supported by Canadian Institutes of Health Research grant FDN-143202.

AUTHOR CONTRIBUTIONS

Conceptualization, J.W., M.M., B.H., and S.G. Formal analysis, J.W., J.P., A.L., M.Y., and M.M. Investigation, J.W., J.P., M.M., and S.G. Resources, B.H., S.A., J.N., and S.G. Writing – original draft, J.W., M.M., and S.G. Writing – review & editing, J.W., M.M., S.A., B.H., J.N., and S.G. Supervision, S.G.

DECLARATION OF INTERESTS

The authors declare no competing interests.

Received: June 24, 2021
Revised: July 21, 2021
Accepted: December 7, 2021
Published: January 4, 2022

REFERENCES

Aggor, F.E.Y., Break, T.J., Trevejo-Nuñez, G., Whibley, N., Coleman, B.M., Bailey, R.D., Kaplan, D.H., Naglik, J.R., Shan, W., Shetty, A.C., et al. (2020). Oral epithelial IL-22/STAT3 signaling licenses IL-17-mediated immunity to oral mucosal candidiasis. Sci. Immunol. *5*, eaba0570.

Allert, S., Förster, T.M., Svensson, C.-M., Richardson, J.P., Pawlik, T., Hebecker, B., Rudolphi, S., Juraschitz, M., Schaller, M., Blagojevic, M., et al. (2018). Candida albicans-Induced Epithelial Damage Mediates Translocation through Intestinal Barriers. MBio *9*, e00915–e00918.

Babiychuk, E.B., Monastyrskaya, K., Potez, S., and Draeger, A. (2009). Intracellular Ca(2+) operates a switch between repair and lysis of streptolysin O-perforated cells. Cell Death Differ. *16*, 1126–1134.

Baracca, A., Sgarbi, G., Solaini, G., and Lenaz, G. (2003). Rhodamine 123 as a probe of mitochondrial membrane potential: evaluation of proton flux through F(0) during ATP synthesis. Biochim. Biophys. Acta *1606*, 137–146.

Bichele, R., Kärner, J., Truusalu, K., Smidt, I., Mändar, R., Conti, H.R., Gaffen, S.L., Peterson, P., Laan, M., and Kisand, K. (2018). IL-22 neutralizing autoantibodies impair fungal clearance in murine oropharyngeal candidiasis model. Eur. J. Immunol. *48*, 464–470.

Blagojevic, M., Camilli, G., Maxson, M., Hube, B., Moyes, D.L., Richardson, J.P., and Naglik, J.R. (2021). Candidalysin triggers epithelial cellular stresses that induce necrotic death. Cell. Microbiol. *23*, e13371.

Bleck, M., Itano, M.S., Johnson, D.S., Thomas, V.K., North, A.J., Bieniasz, P.D., and Simon, S.M. (2014). Temporal and spatial organization of ESCRT protein recruitment during HIV-1 budding. Proc. Natl. Acad. Sci. USA *111*, 12211–12216.

Bohdanowicz, M., Balkin, D.M., De Camilli, P., and Grinstein, S. (2012). Recruitment of OCRL and Inpp5B to phagosomes by Rab5 and APPL1 depletes phosphoinositides and attenuates Akt signaling. Mol. Biol. Cell *23*, 176–187.

Botelho, R.J., Teruel, M., Dierckman, R., Anderson, R., Wells, A., York, J.D., Meyer, T., and Grinstein, S. (2000). Localized biphasic changes in phosphatidylinositol-4,5-bisphosphate at sites of phagocytosis. J. Cell Biol. *151*, 1353–1368.

Černe, K., Erman, A., and Veranič, P. (2013). Analysis of cytotoxicity of melittin on adherent culture of human endothelial cells reveals advantage of fluorescence microscopy over flow cytometry and haemocytometer assay. Protoplasma *250*, 1131–1137.

Chen, T.W., Wardill, T.J., Sun, Y., Pulver, S.R., Renninger, S.L., Baohan, A., Schreiter, E.R., Kerr, R.A., Orger, M.B., Jayaraman, V., et al. (2013). Ultrasensitive fluorescent proteins for imaging neuronal activity. Nature *499*, 295–300.

Conti, H.R., Shen, F., Nayyar, N., Stocum, E., Sun, J.N., Lindemann, M.J., Ho, A.W., Hai, J.H., Yu, J.J., Jung, J.W., et al. (2009). Th17 cells and IL-17 receptor signaling are essential for mucosal host defense against oral candidiasis. J. Exp. Med. *206*, 299–311.

Dalle, F., Wächtler, B., L'Ollivier, C., Holland, G., Bannert, N., Wilson, D., Labruère, C., Bonnin, A., and Hube, B. (2010). Cellular interactions of Candida albicans with human oral epithelial cells and enterocytes. Cell. Microbiol. *12*, 248–271.

Esteban, A., Popp, M.W., Vyas, V.K., Strijbis, K., Ploegh, H.L., and Fink, G.R. (2011). Fungal recognition is mediated by the association of dectin-1 and galectin-3 in macrophages. Proc. Natl. Acad. Sci. USA *108*, 14270–14275.

Guizetti, J., Schermelleh, L., Mäntler, J., Maar, S., Poser, I., Leonhardt, H., Müller-Reichert, T., and Gerlich, D.W. (2011). Cortical constriction during abscission involves helices of ESCRT-III-dependent filaments. Science *331*, 1616–1620.

Hammond, G.R.V., Machner, M.P., and Balla, T. (2014). A novel probe for phosphatidylinositol 4-phosphate reveals multiple pools beyond the Golgi. J. Cell Biol. *205*, 113–126.

Ho, J., Yang, X., Nikou, S.-A., Kichik, N., Donkin, A., Ponde, N.O., Richardson, J.P., Gratacap, R.L., Archambault, L.S., Zwirner, C.P., et al. (2019). Candidalysin activates innate epithelial immune responses via epidermal growth factor receptor. Nat. Commun. *10*, 2297.

Jia, J., Tarabykina, S., Hansen, C., Berchtold, M., and Cygler, M. (2001). Structure of apoptosis-linked protein ALG-2: insights into Ca2+-induced changes in penta-EF-hand proteins. Structure *9*, 267–275.

Jimenez, A.J., Maiuri, P., Lafaurie-Janvore, J., Divoux, S., Piel, M., and Perez, F. (2014). ESCRT machinery is required for plasma membrane repair. Science *343*, 1247136.

Kanai, F., Liu, H., Field, S.J., Akbary, H., Matsuo, T., Brown, G.E., Cantley, L.C., and Yaffe, M.B. (2001). The PX domains of p47phox and p40phox bind to lipid products of PI(3)K. Nat. Cell Biol. *3*, 675–678.

Keyel, P.A., Loultcheva, L., Roth, R., Salter, R.D., Watkins, S.C., Yokoyama, W.M., and Heuser, J.E. (2011). Streptolysin O clearance through sequestration into blebs that bud passively from the plasma membrane. J. Cell Sci. *124*, 2414–2423.

Kirchner, F.R., Littringer, K., Altmeier, S., Tran, V.D.T., Schönherr, F., Lemberg, C., Pagni, M., Sanglard, D., Joller, N., and LeibundGut-Landmann, S. (2019). Persistence of *Candida albicans* in the Oral Mucosa Induces a Curbed Inflammatory Host Response That Is Independent of Immunosuppression. Front. Immunol. *10*, 330.

Kumamoto, C.A., Gresnigt, M.S., and Hube, B. (2020). The gut, the bad and the harmless: Candida albicans as a commensal and opportunistic pathogen in the intestine. Curr. Opin. Microbiol. *56*, 7–15.

Lechat, J., Pascault, A., Thibaut, D., Le Borgne, R., Verbavatz, J.-M., and Weiner, A. (2021). Trans-cellular tunnels induced by the fungal pathogen Candida albicans facilitate invasion through successive epithelial cells without host damage. bioRxiv. https://doi.org/10.1101/2021.09.09.459426.

Liu, J., Willems, H.M.E., Sansevere, E.A., Allert, S., Barker, K.S., Lowes, D.J., Dixson, A.C., Xu, Z., Miao, J., DeJarnette, C., et al. (2021). A variant ECE1 allele contributes to reduced pathogenicity of Candida albicans during vulvovaginal candidiasis. PLoS Pathog. *17*, e1009884.

MacCallum, D.M., Castillo, L., Nather, K., Munro, C.A., Brown, A.J.P., Gow, N.A.R., and Odds, F.C. (2009). Property differences among the four major Candida albicans strain clades. Eukaryot. Cell *8*, 373–387.

Martinez, I., Chakrabarti, S., Hellevik, T., Morehead, J., Fowler, K., and Andrews, N.W. (2000). Synaptotagmin VII regulates Ca(2+)-dependent exocytosis of lysosomes in fibroblasts. J. Cell Biol. *148*, 1141–1149.

Maxson, M.E., Naj, X., O'Meara, T.R., Plumb, J.D., Cowen, L.E., and Grinstein, S. (2018). Integrin-based diffusion barrier separates membrane domains enabling the formation of microbiostatic frustrated phagosomes. eLife *7*, e34798.

Mayer, F.L., Wilson, D., and Hube, B. (2013). Candida albicans pathogenicity mechanisms. Virulence *4*, 119–128.

Mogavero, S., Sauer, F.M., Brunke, S., Allert, S., Schulz, D., Wisgott, S., Jablonowski, N., Elshafee, O., Krüger, T., Kniemeyer, O., et al. (2021a). Candidalysin delivery to the invasion pocket is critical for host epithelial damage induced by Candida albicans. Cell. Microbiol. *23*, e13378.

Moyes, D.L., Runglall, M., Murciano, C., Shen, C., Nayar, D., Thavaraj, S., Kohli, A., Islam, A., Mora-Montes, H., Challacombe, S.J., and Naglik, J.R. (2010). A biphasic innate immune MAPK response discriminates between the yeast and hyphal forms of Candida albicans in epithelial cells. Cell Host Microbe *8*, 225–235.

Moyes, D.L., Richardson, J.P., and Naglik, J.R. (2015). Candida albicans-epithelial interactions and pathogenicity mechanisms: scratching the surface. Virulence *6*, 338–346.

Moyes, D.L., Wilson, D., Richardson, J.P., Mogavero, S., Tang, S.X., Wernecke, J., Höfs, S., Gratacap, R.L., Robbins, J., Runglall, M., et al. (2016). Candidalysin is a fungal peptide toxin critical for mucosal infection. Nature *532*, 64–68.

Naglik, J.R., Gaffen, S.L., and Hube, B. (2019). Candidalysin: discovery and function in Candida albicans infections. Curr. Opin. Microbiol. *52*, 100–109.

Niewerth, M., and Korting, H.C. (2001). Phospholipases of Candida albicans. Mycoses *44*, 361–367.

Paredes-Gamero, E.J., Casaes-Rodrigues, R.L., Moura, G.E.D.D., Domingues, T.M., Buri, M.V., Ferreira, V.H.C., Trindade, E.S., Moreno-Ortega, A.J., Cano-Abad, M.F., Nader, H.B., et al. (2012). Cell-permeable gomesin peptide promotes cell death by intracellular Ca(2+) overload. Mol. Pharm. *9*, 2686–2697.

Perlroth, J., Choi, B., and Spellberg, B. (2007). Nosocomial fungal infections: epidemiology, diagnosis, and treatment. Med. Mycol. *45*, 321–346.

Pfaller, M.A., and Diekema, D.J. (2007). Epidemiology of invasive candidiasis: a persistent public health problem. Clin. Microbiol. Rev. *20*, 133–163.

Rahman, D., Mistry, M., Thavaraj, S., Challacombe, S.J., and Naglik, J.R. (2007). Murine model of concurrent oral and vaginal Candida albicans

colonization to study epithelial host-pathogen interactions. Microbes Infect. *9*, 615–622.

Reddy, A., Caler, E.V., and Andrews, N.W. (2001). Plasma membrane repair is mediated by Ca2+-regulated exocytosis of lysosomes. Cell.

Roberts, R.L., Barbieri, M.A., Ullrich, J., and Stahl, P.D. (2000). Dynamics of rab5 activation in endocytosis and phagocytosis. J. Leukoc. Biol. *68*, 627–632.

Rupniak, H.T., Rowlatt, C., Lane, E.B., Steele, J.G., Trejdosiewicz, L.K., Laskiewicz, B., Povey, S., and Hill, B.T. (1985). Characteristics of four new human cell lines derived from squamous cell carcinomas of the head and neck. J. Natl. Cancer Inst. *75*, 621–635.

Schaller, M., Borelli, C., Korting, H.C., and Hube, B. (2005). Hydrolytic enzymes as virulence factors of Candida albicans. Mycoses *48*, 365–377.

Scheffer, L.L., Sreetama, S.C., Sharma, N., Medikayala, S., Brown, K.J., Defour, A., and Jaiswal, J.K. (2014). Mechanism of Ca^2-triggered ESCRT assembly and regulation of cell membrane repair. Nat. Commun. *5*, 5646.

Schönherr, F.A., Sparber, F., Kirchner, F.R., Guiducci, E., Trautwein-Weidner, K., Gladiator, A., Sertour, N., Hetzel, U., Le, G.T.T., Pavelka, N., et al. (2017). The intraspecies diversity of C. albicans triggers qualitatively and temporally distinct host responses that determine the balance between commensalism and pathogenicity. Mucosal Immunol. *10*, 1335–1350.

Strijbis, K., Tafesse, F.G., Fairn, G.D., Witte, M.D., Dougan, S.K., Watson, N., Spooner, E., Esteban, A., Vyas, V.K., Fink, G.R., et al. (2013). Bruton's Tyrosine Kinase (BTK) and Vav1 contribute to Dectin1-dependent phagocytosis of Candida albicans in macrophages. PLoS Pathog. *9*, e1003446.

Swidergall, M., Khalaji, M., Solis, N.V., Moyes, D.L., Drummond, R.A., Hube, B., Lionakis, M.S., Murdoch, C., Filler, S.G., and Naglik, J.R. (2019). Candidalysin Is Required for Neutrophil Recruitment and Virulence During Systemic Candida albicans Infection. J. Infect. Dis. *220*, 1477–1488.

Teruel, M.N., Blanpied, T.A., Shen, K., Augustine, G.J., and Meyer, T. (1999). A versatile microporation technique for the transfection of cultured CNS neurons. J. Neurosci. Methods *93*, 37–48.

Westman, J., Moran, G., Mogavero, S., Hube, B., and Grinstein, S. (2018). Candida albicans Hyphal Expansion Causes Phagosomal Membrane Damage and Luminal Alkalinization. MBio *9*, e01226-18.

Westman, J., Hube, B., and Fairn, G.D. (2019). Integrity under stress: Host membrane remodelling and damage by fungal pathogens. Cell. Microbiol. *21*, e13016.

Westman, J., Walpole, G.F.W., Kasper, L., Xue, B.Y., Elshafee, O., Hube, B., and Grinstein, S. (2020). Lysosome Fusion Maintains Phagosome Integrity during Fungal Infection. Cell Host Microbe *28*, 798–812.e6.

Witchley, J.N., Penumetcha, P., Abon, N.V., Woolford, C.A., Mitchell, A.P., and Noble, S.M. (2019). Candida albicans Morphogenesis Programs Control the Balance between Gut Commensalism and Invasive Infection. Cell Host Microbe *25*, 432–443.e6.

Wolfmeier, H., Radecke, J., Schoenauer, R., Koeffel, R., Babiychuk, V.S., Drücker, P., Hathaway, L.J., Mitchell, T.J., Zuber, B., Draeger, A., and Babiychuk, E.B. (2016). Active release of pneumolysin prepores and pores by mammalian cells undergoing a Streptococcus pneumoniae attack. Biochim. Biophys. Acta *1860* (*11 Pt A*), 2498–2509.

Zakikhany, K., Naglik, J.R., Schmidt-Westhausen, A., Holland, G., Schaller, M., and Hube, B. (2007). In vivo transcript profiling of Candida albicans identifies a gene essential for interepithelial dissemination. Cell. Microbiol. *9*, 2938–2954.

STAR★METHODS

KEY RESOURCES TABLE

REAGENT or RESOURCE	SOURCE	IDENTIFIER
**Antibodies**		
Rabbit anti-Candida albicans IgG	Acris	Cat#BP1006; RRID:AB_973716
Donkey anti-rabbit IgG Alexa Fluor 647	Jackson ImmunoResearch	Cat#711-605-152; RRID: AB_2492288
Donkey anti-rabbit IgG Alexa Fluor 488	Jackson ImmunoResearch	Cat#711-545-152; RRID: AB_2313584
Donkey anti-mouse IgG Alexa Fluor 488	Jackson ImmunoResearch	Cat#712-545-150; RRID: AB_2340846
Rat anti-LAMP-1 hybridoma	Developmental Studies Hybridoma Bank	Cat#H4A3-s; RRID: AB_528127
Rabbit anti-ALG-2	Proteintech	Cat#12303-1-AP; RRID:AB_2162459
Mouse anti-vinculin	Millipore	Cat#MAB3574; RRID:AB_2304338
Rabbit anti-CHMP4B	Novus Biologicals	Cat#NBP1-91782; RRID:AB_11020392
Rabbit anti-ALIX	Millipore	Cat#ABC40; RRID:AB_11213660
Llama CalF1 nanobody (raised against isolated candidalysin)	(Mogavero et al., 2021)	N/A
Llama CalH1 nanobody (raised against synthetic candidalysin)	(Mogavero et al., 2021)	N/A
**Bacterial and virus strains**		
Candida albicans strain SC5314+BFP (Isogenic wild type)	(Strijbis et al., 2013)	N/A
C. albicans strain BWP17+pCIP30 (Isogenic wild type)	(Zakikhany et al., 2007)	N/A
C. albicans strain BWP17+pCIP30+RFP (Isogenic wild type)	(Westman et al., 2020)	N/A
C. albicans strain BWP17+pCIP30+GFP (Isogenic wild type)	(Westman et al., 2020)	N/A
C. albicans strain *ece1Δ/Δ* (ECE1 deletion strain)	(Moyes et al., 2016)	N/A
C. albicans strain *ece1Δ/Δ* + $ECE1_{\Delta 184\text{-}279}$ (candidalysin deletion strain)		N/A
C. albicans clinical isolate 529L (oral candidiasis)	(Rahman et al., 2007)	N/A
C. albicans clinical isolate 101 (healthy volunteer)	(Schönherr et al., 2017)	N/A
C. albicans clinical isolate Hun96	(MacCallum et al., 2009)	N/A
**Chemicals, peptides, and recombinant proteins**		
EGTA	Bio-World	Cat#40120370; CAS 67-42-5
Thapsigargin	Sigma-Aldrich	Cat#T9033; CAS 67526-95-8
Paraformaldehyde	Electron Microscopy Sciences	Cat#15710; CAS 30525-89-4
Triton X-100	Fisher Scientific	Cat#BP151-500; CAS 9002-93-1
FuGENE HD	Promega	Cat#E2311
Calcofluor White Stain	Sigma-Aldrich	Cat#18909
4′,6-diamidino-2-phenylindole dihydrochloride (DAPI)	Thermo Fisher Scientific	Cat#D1306
Propidium iodide	Sigma-Aldrich	Cat#P4170; CAS 25535-16-4
FM4-64	Invitrogen	Cat#T13320; CAS 162112-35-8
Rhodamine 123	Sigma-Aldrich	Cat# R8004; CAS 62669-70-9
Concanavalin A-647	Thermo Fisher Scientific	Cat#C21421
Albumin	BioShop	Cat#ALB001
Antibiotic-Antimycotic Solution	Wisent Bio Products	Cat#450-115-EL
Amersham ECL Prime Western Blotting Detection Reagent	GE Healthcare	Cat#RPN2232

(*Continued on next page*)

Continued		
REAGENT or RESOURCE	SOURCE	IDENTIFIER
Ethylenediaminetetraacetic acid (EDTA)	Thermo Fisher Scientific	Cat#SS412-1
Recombinant Human IL-22	Peprotech	Cat#200-22
Ece1-III, candidalysin	Peptide Protein Research	N/A
Ece1-VIIa control peptide	Peptide Protein Research	N/A
siRNA against ALG-2	Thermo Fisher Scientific	Cat#1299001(HSS115207)
siRNA scramble peptide	Dharmacon	Cat#D-001810-10-20
Experimental models: Cell lines		
Human TR146 cells	(ECACC 10032305)	N/A
Mouse RAW264.7 (male)	ATCC	Cat#TIB-71
Mouse RAW264.7-Dectin-1-LPETG-3 × HA (male)	(Esteban et al., 2011)	N/A
Recombinant DNA		
LAMP-1-GFP	(Reddy et al., 2001)	Addgene Plasmid 16290
Rab5-GFP	(Bohdanowicz et al., 2012)	Addgene Plasmid 35140
pGP-CMV-GCaMP6s	(Chen et al., 2013)	Addgene Plasmid 40753
CHMP2a-GFP	(Guizetti et al., 2011)	Addgene Plasmid 31805
CHMP4B-mCherry	(Bleck et al., 2014)	Addgene Plasmid 116923
CHMP6-GFP	(Guizetti et al., 2011)	Addgene Plasmid 31806
PM-RFP	(Teruel et al., 1999)	N/A
PX-GFP	(Kanai et al., 2001)	N/A
GFP-2xP4M-SidM	(Hammond et al., 2014)	N/A
PLC∂- PH-GFP	(Botelho et al., 2000)	N/A
Software and algorithms		
Volocity 6.3	Perkin Elmer	https://www.perkinelmer.com/; RRID:SCR_002668
GraphPad Prism 8	GraphPad Software	https://www.graphpad.com/; RRID:SCR_002798
ImageJ software (Fiji v. 2.1.0-rc-65/1.53w)	NIH	https://imagej.net/; RRID:SCR_003070
Imaris software 9.5.1	Oxford Instruments	https://imaris.oxinst.com/; RRID:SCR_007370
QUARK	Zhang Group, University of Michigan	https://zhanglab.ccmb.med.umich.edu/QUARK/; RID:SCR_018777
PyMOL	Schrödinger	http://www.pymol.org RRID:SCR_000305

RESOURCE AVAILABILITY

Lead contact

Further information and requests for resources and reagents should be directed to and will be fulfilled by the lead contact, Sergio Grinstein (sergio.grinstein@sickkids.ca).

Materials availability

All unique/stable reagents generated in this study are available from the lead contact upon request.

Data and code availability

All data reported in this paper will be shared by the lead contact upon request. This paper does not report original code. Any additional information required to reanalyze the data reported in this paper is available from the lead contact upon request.

EXPERIMENTAL MODEL AND SUBJECT DETAILS

Candida albicans strains and cultivation

C. albicans strains included the prototrophic wild-type strains BWP17-pClp30 (Zakikhany et al., 2007) and its parental strain SC5314, the *C. albicans* reporter strains BWP17 + pClp30 + RFP (Westman et al., 2020), BWP17 + pClp30 + GFP (Westman et al., 2020), and SC5314 + BFP (Strijbis et al., 2013). The clinical strains 529L (Rahman et al., 2007), 101 (Schönherr et al., 2017), and Hun96

(MacCallum et al., 2009). An *ECE1* deletion strain (*ece1*Δ/Δ) (Moyes et al., 2016) and lastly an *ece1*Δ/Δ + *ECE1*$_{\Delta184\text{-}279}$ strain (Moyes et al., 2016). *C. albicans* were grown at 30°C overnight while shaking in YPD medium (BD Biosciences, 1% yeast extract, 2% peptone, 2% dextrose). Overnight *C. albicans* cultures were diluted to OD_{600nm} 0.1 in YPD broth and grown at 30°C until OD_{600nm} 1.0. Yeast aggregates were dispersed using a 27-gauge needle prior to infection.

Cell lines

Experiments were carried out using the TR146 human oral epithelial cell line (Rupniak et al., 1985). TR146 cells were grown at 37°C in an air-CO_2 5% environment in medium DMEM/F12 (Wisent Inc.) supplemented with 10% (vol/vol) heat-inactivated fetal bovine serum (FBS, Gibco). Murine RAW264.7-Dectin-1-LPETG-3 × HA cells (Esteban et al., 2011) were grown at 37°C and 5% CO_2 in RPMI medium (Wisent, Inc.) supplemented with 10% (vol/vol) heat-inactivated FBS.

METHOD DETAILS

Plasmids and reagents

The following plasmids were described previously: LAMP1-GFP (Martinez et al., 2000; Reddy et al., 2001), Rab5-GFP (Roberts et al., 2000; Bohdanowicz et al., 2012), pGP-CMV-GCaMP6s (Chen et al., 2013), PLC∂- PH-GFP (Botelho et al., 2000), PM-RFP (Teruel et al., 1999), PX-GFP (Kanai et al., 2001), GFP-2xP4M-SidM (Hammond et al., 2014), CHMP2a-GFP (Guizetti et al., 2011), CHMP4B-mCherry (Bleck et al., 2014), CHMP6-GFP (Guizetti et al., 2011).

Primary antibodies were purchased from the following vendors: anti-*C. albicans* (catalogue #BP1006, Acris), anti-LAMP-1 (catalogue #H4A3-s, Developmental Studies Hybridoma Bank), anti-ALIX (catalogue #ABC40, Millipore), anti-ALG-2 (catalogue #12303-1-AP, Proteintech), anti-Vinculin (catalogue #MAB3574, Millipore). Anti-rabbit secondary antibodies conjugated with Alexa Fluor 488 and Alexa Fluor 647 were purchased from Jackson ImmunoResearch Labs. Anti-candidalysin VHH nanobodies CaLH1 and CaLF1, produced by QVQ B.V. (Utrecht, The Netherlands) (Mogavero et al., 2021), were directly labeled by conjugation with Alexa 488 or Alexa 568 NHS-esters (Invitrogen), following manufacturer's instructions.

Thapsigargin, calcofluor white stain (CFW, Fluorescent Brightener 28), DAPI, propidium iodide, ionomycin and cresyl violet were from Sigma-Aldrich. FM4-64 and Rhodamine123 were from Invitrogen. Phalloidin, fluorescently-conjugated ConA, and Triton X-100 were from ThermoFisher Scientific. Acti-stain from Cytoskeleton, Inc.. Paraformaldehyde was from Electron Microscopy Sciences. Synthetic candidalysin (SIIGIIMGILGNIPQVIQIIMSIVKAFKGNK) and the control peptide Ece1-VIIa (DGLEDFLDELLQRLPQLIT) were from Peptide Protein Research. Human recombinant IL-22 was from Peprotech.

DNA transfection

For transient transfection, TR146 cells were plated on 18 mm glass coverslips at 1.5×10^5 cells mL^{-1}, 16-24 h prior to experiments. The cells were transfected at a 3:1 ratio using 1.5 μL Lipofectamine LTX (Invitrogen, Thermo Fisher Scientific), 0.5 μg DNA and 0.5 μL of PLUS reagent (Invitrogen, Thermo Fisher Scientific) per well, for 4 h in Opti-MEM medium (Gibco, Thermo Fisher Scientific). Following this, the medium was changed to DMEM/F12 containing 10% heat-inactivated FBS, and cells were used for experiments 16 h after transfection.

Epithelial invasion assay

TR146 cells were plated on 18 mm glass coverslips at 1.5×10^5 cells mL^{-1} in DMEM/F12 containing 10% FBS for 16 to 48 h. The day of the experiment, medium was changed to a minimal medium (140 mM NaCl, 3 mM KCl, 1 mM $CaCl_2$, 1 mM $MgCl_2$, 15 mM HEPES, 5 mM glucose, pH 7.4), and *C. albicans* strains added to monolayers at MOI of 0.25 to 0.5. After addition of *C. albicans*, plates were centrifuged for 1 min at 1500 rpm, then incubated at 37°C under 5% CO_2 for up to 5 h. Unbound *C. albicans* cells were washed 3 times in PBS and samples were fixed with 4% paraformaldehyde (PFA). External *C. albicans* were labeled for 20 min at room temperature using a solution of 5 $\mu g.mL^{-1}$ fluorescent conjugated concanavalin A. In some cases, *C. albicans* were stained with 10 $\mu g.mL^{-1}$ calcofluor white. Candidalysin was visualized staining with CalH1 (1:100) or CalF1 (1:500) antibodies, as indicated in the text.

To stain actin filaments, cells were permeabilized 5 min with 0.1% Triton X-100 and incubated 30 min with a 1:1000 dilution of fluorescent phalloidin or Acti-stain.

Cytosolic Ca^{2+} measurements using pGP-CMV-GCaMP6s

After transfection of TR146 cells with pGP-CMV-GCaMP6s for 16 h, cells were monitored by time-lapse confocal microscopy for Ca^{2+} transients in the minimal medium with or without 1 mM Ca^{2+}. 30 μM candidalysin or Ece1-VIIa was added at time point 0 s and (Ca^{2+}) recorded every 20 s for 12 min. Thapsigargin was added to a final concentration of 500 nM at the 12 min mark and the GCaMP6s fluorescence intensity was recorded for an additional 3 min. For TR146 cells that were silenced for ALG-2 or control siRNA, fluorescence intensity was measured for each cell every minute for 12 min. Column statistics was performed on all of the GCaMP6s fluorescence intensity values to calculate the standard deviation of each cell.

Visualization of TR146 membrane blebbing in response to candidalysin

TR146 cells were grown on glass coverslips as described above, and incubated with 30 μM candidalysin, 30 μM Ece1-VIIa, or infected with *C. albicans* yeast. At indicated time points, samples were washed in ice-cold PBS and incubated 10 min at 10°C to arrest endocytosis. Monolayers were stained for 5 min with 20 μM FM4-64 in cold PBS and imaged immediately for FM4-64-positive membrane blebs. In cases were TR146 cells were infected with *C. albicans* strains, external *C. albicans* were labeled adding 5 μg.mL^{-1} of fluorescent ConA during the initial 10 min of incubation at 10°C, and all *C. albicans* stained with 10 μg.mL^{-1} calcofluor during the FM4-64 staining step.

ALG-2 immunostaining

TR146 cells were grown on glass coverslips as described above, and incubated with 30 μM candidalysin, 30 μM Ece1-VIIa, or infected with *C. albicans* yeast. At indicated time points, samples were washed in PBS and fixed with 4% PFA for 20 min at room temperature. Samples were washed twice in PBS and the PM was labeled with fluorescent ConA (1:1000 in PBS, 20 min, room temperature). After two washes in PBS, samples were permeabilized with 0.2% Triton X-100 (15 min, room temperature) and incubated with blocking buffer (2% FBS, 2% BSA) for 60 min at room temperature. Samples were immunostained with rabbit anti-ALG-2 antibody (1:100 in blocking buffer, 60 min) and a fluorescent secondary antibody (1:1000 in blocking buffer, 60 min).

siRNA-mediated silencing and immunoblotting of ALG-2

ALG2-targeting stealth RNAi (siRNA) were purchased from ThermoFisher Scientific. Non targeting Stealth siRNAs were purchased from Dharmacon. siRNA delivery was performed using the Neon transfection system (Life Technologies, ThermoFisher Scientific). TR146 cells were resuspended to 5 × 10^{6} cells mL^{-1}, and 100 μL of suspension mixed with 200 pmol control or ALG-2 siRNA. Electroporation was done using two 20 ms pulses of 1300 V. After electroporation, cells were immediately transferred to DMEM/F12 containing 10% heat-inactivated FBS, before seeding on coverslips at concentration of 2.5 × 10^{6} cells mL^{-1}. siRNA-treated cells were used for experiments 48 h after electroporation. In some cases, after 24 h, cells were transiently transfected with mammalian expression vectors as described.

siRNA-mediated knockdown was confirmed at 48 h by immunoblotting. siRNA-treated cells were lysed in Laemmli buffer (Bio-Rad). Lysates were separated by SDS-PAGE, followed by transfer to a polyvinylidene difluoride membrane. The membrane was blocked in TBS containing 1% skim milk and 0.05% Tween 20 for 30 min at room temperature, followed by primary antibody staining for ALG-2 (1:1000 in 1% skim milk and 0.05% Tween 20 for 60 min) or the loading control Vinculin (1:1000 in 1% skim milk and 0.05% Tween 20 for 60 min) for 1 h at room temperature, in blocking buffer. After washing the membrane in TBS containing 0.05% Tween 20, samples were incubated 30 min at room temperature with an HRP-conjugated secondary antibody at 1:5000 dilution. Blots were visualized using BioRad ChemiDoc MP Imaging System and Image Lab software 5.2.1.

Cell damage assays using siRNA-treated TR146 cells

TR146 cells were grown as described above and silenced using electroporation as described above. For some experiments, monolayers were first washed with 1X PBS and incubated in minimal medium with 10 μg.mL^{-1} rhodamine123 for 15 min at 37°C to label mitochondria (Baracca et al., 2003). Following this, monolayers were washed three times with 1X PBS and incubated for 60 min in the presence or absence of 10 μM candidalysin. The cells were then washed in ice-cold PBS and incubated 10 min at 10°C, then stained for 5 min in cold minimal medium containing 5 μg.mL^{-1} DAPI and 20 μM FM4-64 to visualize cell death and blebbing, respectively, and imaged immediately by confocal microscopy.

Alternatively, TR146 cells silenced with ALG-2 siRNA or scrambled siRNA were incubated with 10 μM candidalysin in minimal medium at 37°C. Following incubation, the nuclei of dead cells were stained with 20 μM propidium iodide, permeabilized with 0.2% Triton X-100, and counter-stained for total nuclei with SYTOX Green. Candidalysin-induced cell death was calculated as (propidium iodide-positive cells/SYTOX Green-positive cells) x 100.

LAMP-1 immunostaining of invasion pockets

TR146 cells were grown on glass coverslips and infected with *C. albicans* as described above. At indicated time points, samples were washed in PBS and fixed with 4% PFA for 15 min at room temperature. Samples were washed extensively in PBS and blocked for 60 min with a blocking buffer containing 2% BSA and 2% FBS, immunostained with anti-LAMP-1 (1:100 in blocking buffer, 60 min) and a secondary fluorescent antibody (1:1000 in block buffer, 60 min).

Exofacial LAMP-1 immunostaining

TR146 cells were grown on glass coverslips as described above and incubated with 30 μM candidalysin or the control peptide Ece1-VIIa. At indicated time points, cells were washed in PBS and fixed with 4% PFA. Cells were washed in PBS and incubated for 60 min in blocking buffer containing 2% BSA and 2% FBS. Samples were immunostained with anti-LAMP-1 hybridoma (1:100 in blocking buffer, 60 min) and a fluorescent secondary antibody (1:1000 in blocking buffer, 60 min). The plasma membrane of TR146 cells was labeled with fluorescent ConA (1:1000 in blocking buffer). After immunostaining, samples were permeabilized in 0.1% Triton X-100 and cell nuclei were labeled with 20 μM propidium iodide.

Visualization of TR146 membrane debris and membrane blebs

TR146 cells were grown on glass coverslips as described above, and incubated with 10 or 30 μM candidalysin, or infected with *C. albicans* yeast as indicated. In some cases, after treatments, TR146 cells were fixed for 10min in 4% PFA, washed with PBS, and stained with ConA (1:1000) and CaLH1 nanobody (1:500) for 20 min at room temperature. In other cases, monolayers were stained for 5 min with 20 μM FM4-64 in cold PBS and imaged immediately by time-lapse microscopy at 37°C for the formation of FM4-64-positive membrane blebs. For experiments where TR146 cells were infected with *C. albicans* clinical isolates, after 4 h infection, samples were washed in ice-cold PBS and incubated 10 min at 10°C to arrest endocytosis. External *C. albicans* were labeled adding 5 μg.mL^{-1} of fluorescent ConA during the initial 10 min of incubation at 10°C, and all *C. albicans* stained with 10 μg.mL^{-1} calcofluor during the 20 μM FM4-64 staining step.

Candidalysin peptide structure prediction and modeling

C-QUARK (https://zhanggroup.org/C-QUARK/) was used to generate *ab initio* structure predictions for the candidalysin peptides sequences available for *C. albicans* isolates SC5314 (Moyes et al., 2016) and 529L (Liu et al., 2021). Generated PDB files were then modeled and annotated in PyMOL (Schrödinger).

Immunoblotting of CHMP4B and calculations for cell viability experiments

TR146 cells were plated on 18 mm glass coverslips at 1.5 × 10^5 cells mL^{-1} in DMEM/F12 containing 10% FBS for 16 to 48 h. TR146 cells transfected with CHMP4B-mCherry were lysed in RIPA buffer containing protease inhibitors and diluted in 4x Laemmli buffer (Bio-Rad). Lysates were separated by SDS-PAGE, followed by transfer to a polyvinylidene difluoride membrane. The membrane was blocked in TBS containing 1% skim milk and 0.05% Tween 20 for 60 min at room temperature, followed by primary antibody staining for CHMP4B (1:300 in 1% skim milk and 0.05% Tween 20) or the loading control Vinculin (1:1000 in 1% skim milk and 0.05% Tween 20) for 16 h at 4°C. After washing the membrane in TBS containing 0.05% Tween 20, samples were incubated 60 min at room temperature with an HRP-conjugated secondary antibody at 1:5000 dilution. Blots were visualized using BioRad ChemiDoc MP Imaging System and Image Lab software 5.2.1. The ratio between CHMP4B-mCh and endogenous CHMP4B were calculated by comparing the intensity of the two bands on the same PVDF membrane. To calculate CHMP4B-mCh and CHMP4B protein levels, the immunoblotting measurements were normalized to exposure times and transfection efficiency of CHMP4B-mCh.

Effect of IL-22 on viability of TR146 cells

TR146 cells were grown on glass coverslips as described above and incubated with human recombinant IL-22 or vehicle for 48h.TR146 cells stimulated with IL-22 or vehicle were incubated with 30 μM candidalysin in minimal medium at 37°C. Following incubation, the nuclei of dead cells were stained with 20 μM propidium iodide, permeabilized with 0.2% Triton X-100, and counter-stained for total nuclei with SYTOX Green. Candidalysin-induced cell death was calculated as (propidium iodide-positive cells/SYTOX Green-positive cells) x 100.

Measuring the acidity of *C. albicans*-containing invasion pockets

TR146 cells were plated on 18 mm glass coverslips at 1.5 × 10^5 cells mL^{-1} in DMEM/F12 containing 10% FBS for 16 to 48 h. The day of the experiment, medium was changed to a minimal medium (140 mM NaCl, 3 mM KCl, 1 mM $CaCl_2$, 1 mM $MgCl_2$, 15 mM HEPES, 5 mM glucose, pH 7.4), and *C. albicans* strains added to monolayers at MOI of 0.25–0.5. After addition of *C. albicans*, plates were centrifuged for 1 min at 1500 rpm, then incubated at 37°C under 5% CO_2 for up to 5 h. External *C. albicans* were labeled for 20 min at room temperature using a solution of 5 μg.mL^{-1} fluorescent conjugated concanavalin A. The acidity of *C. albicans*-containing invasion pockets was assessed by incubating TR146 cells infected with the wild-type fungus with cresyl violet for 2 min prior to spinning-disk confocal imaging.

Microscopy

Confocal images were acquired using a spinning disk system (WaveFX; Quorum Technologies Inc.). The instrument consists of a microscope (Axiovert 200M; Zeiss), scanning unit (CSU10; Yokogawa Electric Corporation), electron-multiplied charge-coupled device (C9100-13; Hamamatsu Photonics), five-line (405-, 443-, 491-, 561-, and 655-nm) laser module (Spectral Applied Research), and filter wheel (MAC5000; Ludl) and is operated by Volocity software version 6.3. Confocal images were acquired using a 63×/1.4-N.A. oil objective (Zeiss) coupled to an additional 1.5x magnifying lens and the appropriate emission filter. Cells imaged live were maintained at 37°C using an environmental chamber (Live Cell Instruments).

Epifluorescence images were acquired using an EVOS M5000 Imaging System (Thermo Fisher Scientific). The instrument consists of a high-sensitivity 3.2 MP (2048 × 1536) CMOS monochrome camera with 3.45 μm pixel resolution, three-position chamber (470/525-, 531/593-, 585/624-nm) LED light cubes, and phase contrast imaging mode. Images were acquired using a long working distance 10×/0.3-N.A air objective (Invitrogen). EVOS M5000 images were analyzed using ImageJ (Fiji 2.1.0/1.53c).

QUANTIFICATION AND STATISTICAL ANALYSIS

Image analysis and statistics

Image handling, quantification, and analysis of fluorescence images were performed using Volocity 6.3 software (Quorum Technologies) and ImageJ (Fiji v. 2.1.0/1.53c) software. The figure legends describe the exact number of independent replicates that were analyzed in each experiment. A total of at least 30 cells were quantified for each condition, and the data presented includes all measured data points for all experiments, including means ± standard error of the mean (SEM) or bar plots. Statistical analysis was performed using the means of 3 to 5 individual experiments, and statistical significance was determined using unpaired *t* test and one-way analysis of variance (ANOVA) (Tukey's test or Dunnett's test) with Prism 7 (GraphPad Software), with $p < 0.05$ considered significant.

Cell Reports, Volume *38*

Supplemental information

Calcium-dependent ESCRT recruitment and lysosome exocytosis maintain epithelial integrity during *Candida albicans* invasion

Johannes Westman, Jonathan Plumb, Anna Licht, Mabel Yang, Stefanie Allert, Julian R. Naglik, Bernhard Hube, Sergio Grinstein, and Michelle E. Maxson

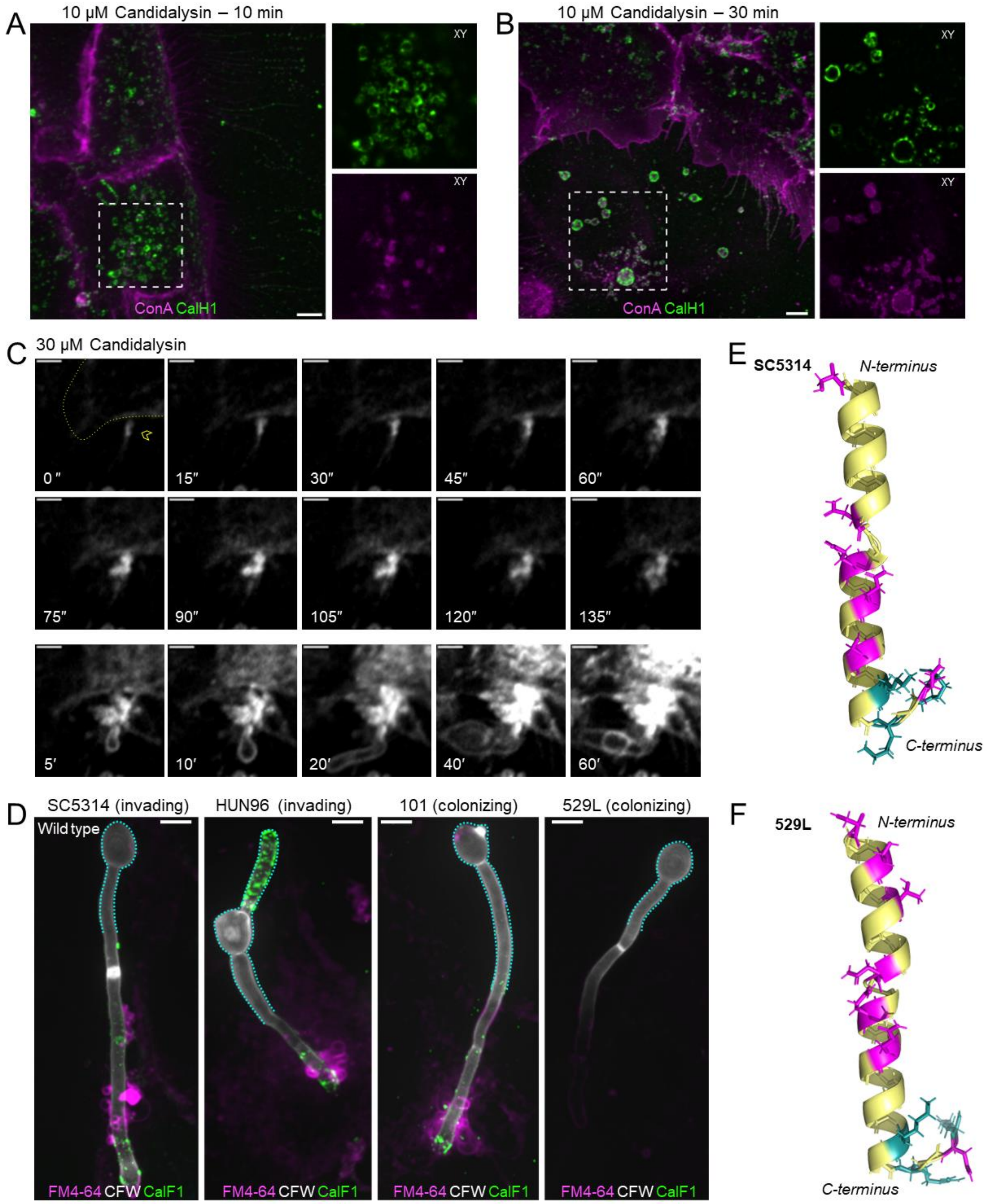

A
10 μM Candidalysin – 10 min
XY
XY
ConA CalH1
B
10 μM Candidalysin – 30 min
XY
XY
ConA CalH1
C
30 μM Candidalysin
0″
15″
30″
45″
60″
75″
90″
105″
120″
135″
5′
10′
20′
40′
60′
D
SC5314 (invading)
HUN96 (invading)
101 (colonizing)
529L (colonizing)
Wild type
FM4-64 CFW CalF1
FM4-64 CFW CalF1
FM4-64 CFW CalF1
FM4-64 CFW CalF1
E
SC5314
N-terminus
C-terminus
F
529L
N-terminus
C-terminus

Figure S1. Candidalysin induces epithelial plasma membrane blebbing, related to Figure 4.

Confocal micrographs of TR146 treated with 10 µM candidalysin peptide for (A) 10 min or (B) 30 min. After treatments, non-permeablized cells were stained for the PM and PM-derived blebs/debris using ConA, and for the localization of candidalysin using the CaLH1 nanobody. Side panels are *XY* slices showing individual CalF1 (green) and ConA (magenta) channels, for areas marked by the dotted boxes. (C) TR146 treated with incubated with 20 µM FM4-64 and cell Z-stacks imaged by time-lapse microscopy. After 1 min of imaging, 30 µM candidalysin peptide was added, and cells imaged for a total time of 90 min. Cell plasma membrane and location of bleb formation is marked in the first panel by the dotted yellow line and yellow arrowhead, respectively. Panels are extended focus projections. (D) Representative confocal micrographs of TR146 invasion pockets induced by *C. albicans* clinical isolates SC5314, HUN96, 101 and 529L. Bleb formation and candidalysin was assessed by staining non-permeabilized cells with 10 µM FM4-64 and CaLF1 nanobody, respectively. External portions of the isolates, stained with ConA, are indicated by the dotted cyan outlines. All scale bars = 5 µm. Images are representative of at least 3 experiments of each type. Structure predictions for the candidalysin peptides of isolates (E) SC5314 and (F) 529L. Amino acid residues were colored in PyMOL as follows: Hydrophobic– yellow, polar– magenta, and charged– teal.

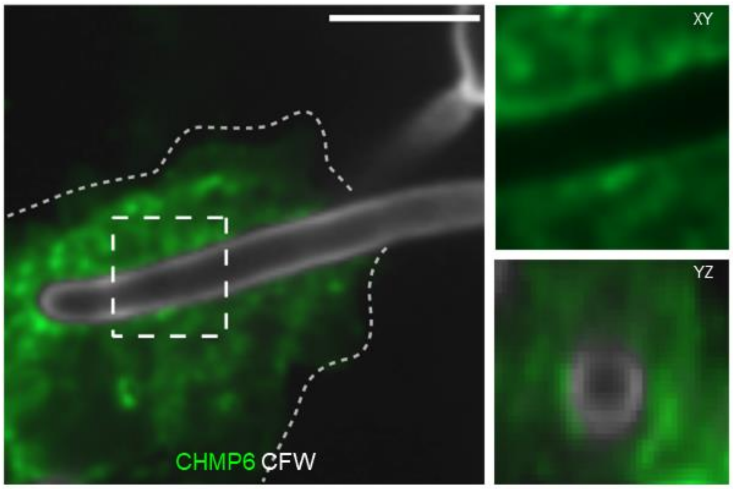

Figure S2. CHMP6 localizes to the invasion pocket membrane, related to Figure 5.

(A) The invasion pocket recruits ESCRT-III component CHMP6. Confocal micrograph of invasion pockets containing wild-type *C. albicans.* TR146 cells were transfected with CHMP6-GFP. Side panels show the XY and YZ slices corresponding to the areas marked by the dashed boxes. Outlines of TR146 cells are indicated by dotted lines. Scale bars = 5 µm.

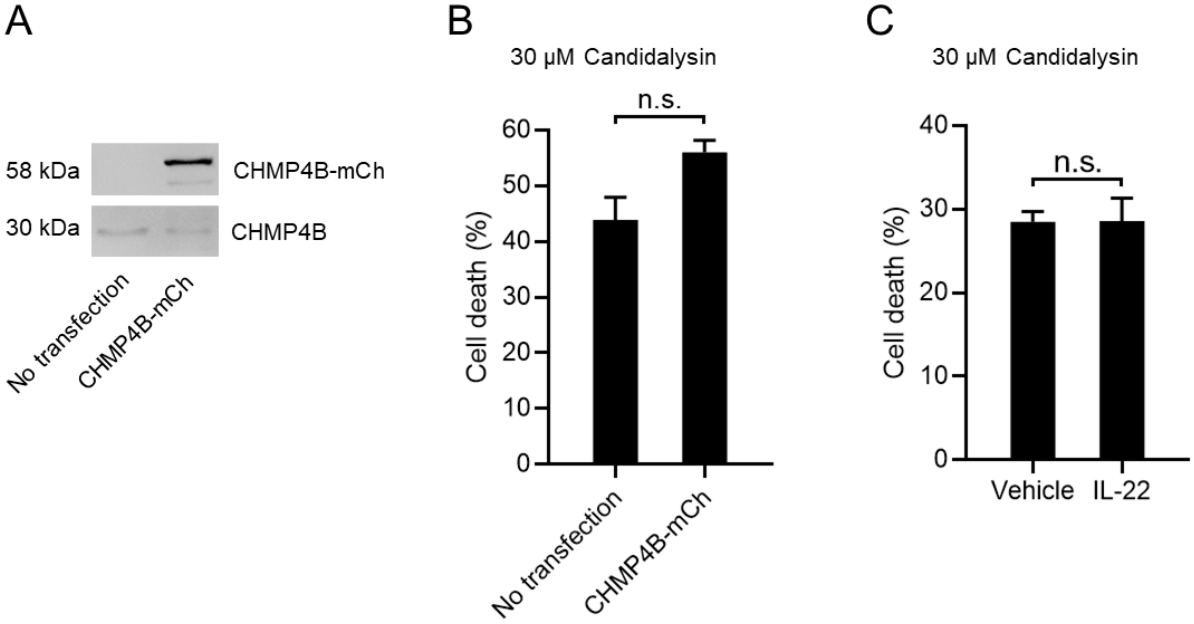

Figure S3. Overexpression of the ESCRT protein CHMP4B does not improve cell viability after treatment with candidalysin, related to Figure 6.

(A) CHMP4B-mCherry was transiently overexpressed in TR146 cell using Lipofectamine LTX. The protein expression of endogenous CHMP4B and CHMP4B-mCherry was measured using different exposure times on the same Western blotting membrane. (B) Quantification of cell death in CHMP4B-mCh transfected TR146 cells and in non-transfected cell. Data are means ± SEM of 3 individual experiments. (C) Human recombinant IL-22 does not improve cell viability after treatment with candidalysin.TR146 cells were incubated with human recombinant 100ng/mL IL-22 for 48 h prior to a treatment with 30 µM candidalysin for 1 h. Cells were incubated with propidium iodide to identify dead cells, fixed in 4% PFA and permeabilized with 0.2% TritonX-100. Lastly, cells were counter-stained with SYTOX Green to visualize all nuclei. Data are means ± SEM of 3 individual experiments.

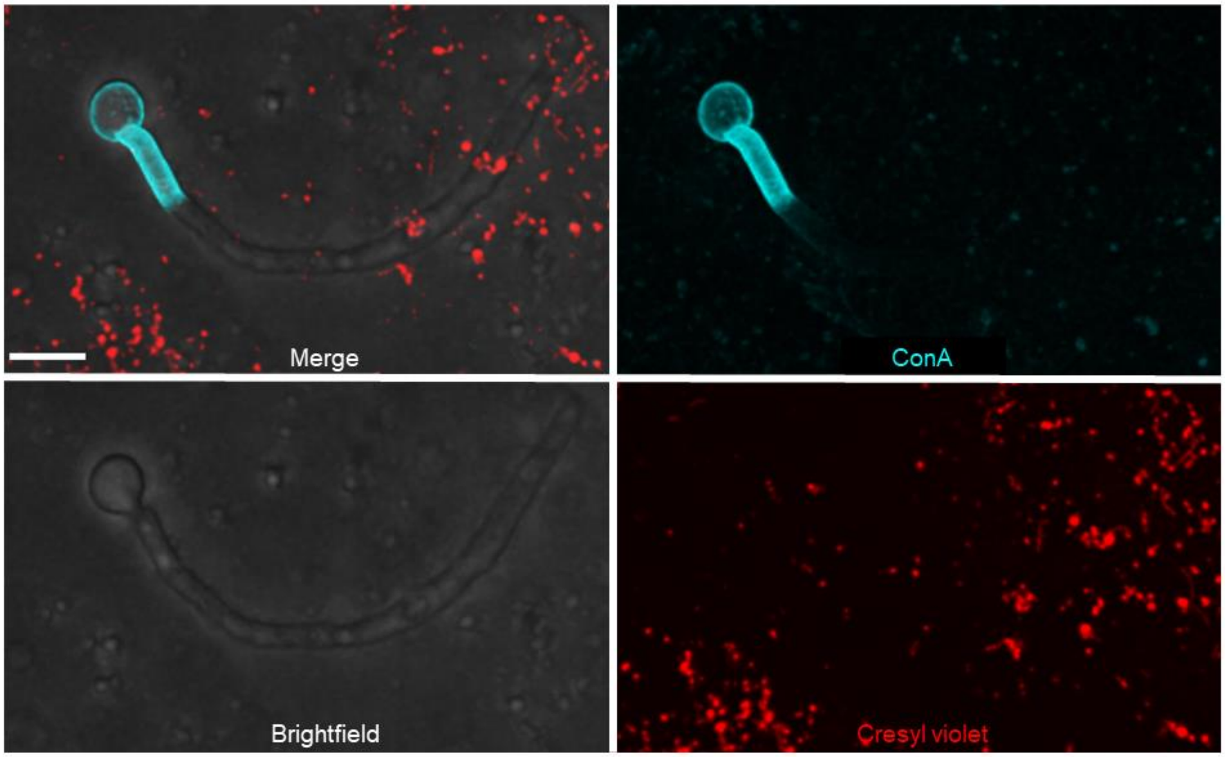

Figure S4. Invasion pockets do not retain the acidotropic dye cresyl violet, related to Figure 7.

The acidity of *C. albicans*-containing invasion pockets was assessed by incubating TR146 cells infected with the wild-type fungus with cresyl violet (red) for 2 min prior to spinning-disk confocal imaging. Extracellular portions of the invading *C. albicans* were stained with ConA (cyan). Scale bar = 5 µm.
